# Obsessive–Compulsive Disorder as an Epiphenomenon of Comorbid Bipolar Disorder? An Updated Systematic Review

**DOI:** 10.3390/jcm13051230

**Published:** 2024-02-21

**Authors:** Renato de Filippis, Andrea Aguglia, Alessandra Costanza, Beatrice Benatti, Valeria Placenti, Eleonora Vai, Edoardo Bruno, Domenico De Berardis, Bernardo Dell’Osso, Umberto Albert, Pasquale De Fazio, Mario Amore, Gianluca Serafini, Nassir S. Ghaemi, Andrea Amerio

**Affiliations:** 1 Psychiatry Unit, Department of Health Sciences, University Magna Graecia of Catanzaro, 88100 Catanzaro, Italy; 2 Department of Neuroscience, Rehabilitation, Ophthalmology, Genetics, Maternal and Child Health (DINOGMI), University of Genoa, 16132 Genoa, Italy; 3 IRCCS Ospedale Policlinico San Martino, 16132 Genoa, Italy; 4 Department of Psychiatry, Faculty of Medicine, University of Geneva (UNIGE), 1205 Geneva, Switzerland; 5 Department of Psychiatry, Faculty of Biomedical Sciences, University of Italian Switzerland (USI) Lugano, 6900 Lugano, Switzerland; 6 Department of Biomedical and Clinical Sciences Luigi Sacco, Luigi Sacco Hospital, University of Milan, 20157 Milan, Italy; 7 NHS, Department of Mental Health, Psychiatric Service for Diagnosis and Treatment, Hospital “G. Mazzini”, 64100 Teramo, Italy; 8 “Aldo Ravelli” Center for Nanotechnology and Neurostimulation, University of Milan, 20122 Milan, Italy; 9 Department of Psychiatry and Behavioral Sciences, Stanford University, Stanford, CA 94305, USA; 10 Department of Medicine, Surgery and Health Sciences, University of Trieste and Department of Mental Health, Azienda Sanitaria Universitaria Giuliano Isontina—ASUGI, 34128 Trieste, Italy; 11 Department of Psychiatry, Tufts University School of Medicine, Boston, MA 02111, USA; 12 Department of Psychiatry, Harvard Medical School, Boston, MA 02115, USA

**Keywords:** biological markers, bipolar disorder, comorbidity, course of illness, epidemiology, heredity, obsessive–compulsive disorder, prevalence, phenomenology, treatment

## Abstract

Background: Bipolar disorder (BD) and obsessive–compulsive disorder (OCD) comorbidity is an emerging condition in psychiatry, with relevant nosological, clinical, and therapeutic implications. Methods: We updated our previous systematic review on epidemiology and standard diagnostic validators (including phenomenology, course of illness, heredity, biological markers, and treatment response) of BD-OCD. Relevant papers published until (and including) 15 October 2023 were identified by searching the electronic databases MEDLINE, Embase, PsychINFO, and Cochrane Library, according to the PRISMA statement (PROSPERO registration number, CRD42021267685). Results: We identified 38 new articles, which added to the previous 64 and raised the total to 102. The lifetime comorbidity prevalence ranged from 0.26 to 27.8% for BD and from 0.3 to 53.3% for OCD. The onset of the two disorders appears to be often overlapping, although the appearance of the primary disorder may influence the outcome. Compared to a single diagnosis, BD-OCD exhibited a distinct pattern of OC symptoms typically following an episodic course, occurring in up to 75% of cases (vs. 3%). Notably, these OC symptoms tended to worsen during depressive episodes (78%) and improve during manic or hypomanic episodes (64%). Similarly, a BD course appears to be chronic in individuals with BD-OCD in comparison to patients without. Additionally, individuals with BD-OCD comorbidity experienced more depressive episodes (mean of 8.9 ± 4.2) compared to those without comorbidity (mean of 4.1 ± 2.7). Conclusions: We found a greater likelihood of antidepressant-induced manic/hypomanic episodes (60% vs. 4.1%), and mood stabilizers with antipsychotic add-ons emerging as a preferred treatment. In line with our previous work, BD-OCD comorbidity encompasses a condition of greater nosological and clinical complexity than individual disorders.

## 1. Introduction

Following the classic formulation by Alvan R. Feinstein, comorbidity should be defined in relation to a specific index condition, as “any distinct additional entity that has existed or may occur during the clinical course of a patient who has the index disease under study” [1]. That is, that two diseases co-occur, more often than not, randomly. Afterwards, the Diagnostic and Statistical Manual of Mental Disorders (DSM) expanded the concept of comorbidity using it to mean the co-occurrence of symptoms [2,3]. Since many DSM diagnoses, especially mood and anxiety disorders, have overlapping symptoms, this use of the term automatically produced many comorbidities [2]. Apparent comorbidity between bipolar disorder (BD) and obsessive-compulsive disorder (OCD) is a common condition in psychiatry with important nosological and therapeutic implications [4,5]. Results from our previous work revealed that the majority of comorbid OCD cases appeared to be secondary to mood episodes [6], with mood stabilization as primary goal in treatment strategies [7]. However, despite recent evidence from the literature [6,8,9], the meaning of BD-OCD comorbidity has not been clarified yet and we cannot say for sure if this common comorbidity represents two separate diseases that cooccur by chance, or a severe subtype of BD or OCD. A diagnosis can be considered valid when one can differentiate it from other diagnoses. In a classic 1970 paper Robins and Guze described criteria to define the validity of a diagnostic construct of interest based on the availability of data on phenomenology, course of illness, heredity, biological markers, and treatment response [10]. Following Robins and Guze’s criteria [10], we systematically updated our previous review [6] to define the nosological validity of comorbid BD-OCD and to provide recommendations for clinicians. 

## 2. Materials and Methods

The study was conducted following the methods recommended by Cochrane Collaboration and documented the process and results in accordance with the last version of the preferred reporting items for systematic reviews and meta-analyses (PRISMA, Berlin, Germany) guidelines [11]. We followed the same search strategy and inclusion/exclusion criteria already applied in our previous systematic review published in 2014 [6], to achieve a high level of internal consistency making the results comparable (PROSPERO registration number CRD42021267685). The PRISMA Checklist is reported in Supplementary Table S1.

### 2.1. Information Sources and Search Strategy

Two authors (RdF and AA) independently searched PubMed, Embase, PsychINFO, and the Cochrane Library databases for articles dealing with BD-OCD comorbidity that were published from 30 March 2013 up to 15 October 2023, without language and time restrictions, using the combination of the following keywords and MeSH terms: ((((((Bipolar disorder) OR BD) OR Bipolar) OR Manic-depressive disorder) OR Manic-depressive) OR Manic) AND (((Obsessive–compulsive disorder) OR Obsessive–compulsive) OR OCD). This search strategy was first developed in PubMed and then adapted for use in the other selected databases (Supplementary Table S2). Reference lists from included articles and previous similar reviews were used to search for additional studies. Additional internet (Google, Menlo Park, CA, USA) searches were also conducted to identify unpublished studies.

### 2.2. Inclusion and Exclusion Criteria

We considered studies that included patients suffering from BD-OCD comorbidities if diagnostic criteria used for both disorders were specified. Among BD study populations, studies that only focused on BD type I (BD-I), BD type II (BD-II), or BD not otherwise specified [12] in comorbidity with OCD defined according to ICD or DSM, or validated diagnostic scales (i.e., SCID: Structured Clinical Interview; CIDI: WHO Composite International Diagnostic Interview; DIS: Diagnostic Interview Schedule; MINI: Mini International Neuropsychiatric Interview; DIGS: Diagnostic Instrument for Genetic Studies; DICA-R: Diagnostic Interview for Children and Adolescents-Revised; SADS-L: Schedule for Affective Disorders and Schizophrenia, Lifetime Version; WASH-U-K-SADS: Washington University in St. Louis Kiddie Schedule for Affective Disorders and Schizophrenia; DIS: Diagnostic Interview Schedule; DIA-X/M-CIDI: Munich Composite International Diagnostic Interview), or clinical records [12,13,14] have been included. Studies that considered subjects with bipolar and obsessive–compulsive spectrum disorders have been also included if diagnostic criteria used were specified [15,16,17]. Participants of both sexes older than 6 years of age have been considered. Studies conducted on subjects with other severe psychiatric diseases or systemic physical comorbidities such as autism spectrum disorders or lupus erythematous have been excluded as non-representative of the study population [18,19]. Both population-based and hospital-based studies have been included. Among hospital-based studies, in-patient, day-hospital, and out-patient subjects populations have been included, while emergency care records have been excluded as considered non-representative. All experimental and observational studies, with retrospective, cross-sectional or longitudinal design have been included. Narrative and systematic reviews, letters to the editor, and book chapters have been excluded as well. Case-report, case series, review, systematic review, animal study, editorial, opinion papers, commentary, study protocol, and study with population or outcome not of interest were excluded. Finally, we excluded any duplicate papers and studies with other adjuvant therapies that interfered with the clinical evaluation of the comorbidity, as well as abstracts and non-English literature.

### 2.3. Outcome Measures

We set the BD-OCD comorbidity prevalence rates and specific confirmative features for a BD-OCD clinical construct as our primary outcomes. Considering comorbidity prevalence rates, we retrieved data on: (1) lifetime prevalence of comorbid OCD in patients primary suffering from BD; and (2) lifetime prevalence of comorbid BD in patients primary suffering from OCD. Therefore, we excluded all studies reporting only data about current prevalence. Regarding confirmative criteria for definition of BD-OCD clinical construct and diagnosis, we used the approach described by Robins and Guze [10], and already applied in our previous work about BD-OCD comorbidity [6], retrieving data on: (1) phenomenology, (2) course of illness, (3) heredity, (4) biological markers, and (5) treatment response. We extracted data according to percentages, odds ratio, effect size, or other result forms, as per authors’ original reports.

### 2.4. Study Selection and Data Extraction

Study selection and data extraction were conducted independently by two authors (RdF and AA) using an ad hoc-developed data extraction spreadsheet. Firstly, the same two authors independently screened title/abstracts for eligibility. Then, the full texts of potentially eligible articles were retrieved, and the same investigators independently scrutinized each study for eligibility. The inconsistencies were overcome with a consensus. The data extraction spreadsheet was modeled on 10 randomly selected papers and modified accordingly.

### 2.5. Quality Assessment

The quality assessment scores assigned to each selected study are reported in Table 1. The same authors who performed data extraction (RdF, AA) independently assessed the quality of the studies included, applying the checklist developed by Downs and Black for both randomized and non-randomized clinical studies [20]. Disagreements by reviewers were resolved via consensus.

## 3. Results

**Figure 1:**
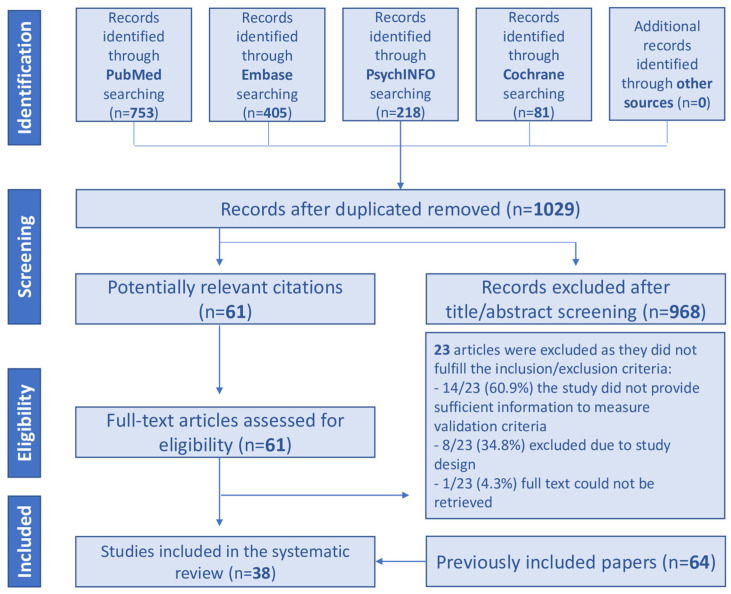
PRISMA flow chart.

Screening and search selection are summarized in Figure 1. After duplicates were removed, 1029 papers were retrieved after searching the selected databases and the listed references of relevant articles. Out of 1029 items, 968 were excluded in the title/abstract screening phase because they did not fulfill inclusion/exclusion criteria. The full text of the remaining 61 papers were reviewed in detail. Finally, 38 studies were included in this systematic review. Then, the articles identified were added to the 64 papers already included in our previous systematic review [6]. Thus, the total number of articles globally considered rose to 102. All previously and new included studies are reported in Supplementary Table S3.

### 3.1. Included Studies

Table 1 summarizes the main characteristics of included studies divided according to the age of the individuals enrolled in the sample (children/adolescents or adults) and setting (population- or hospital-based) [15,16,17,22,23,24,25,26,27,28,29,30,31,32,33,34,35,36,37,38,39,40,41,42,43,44,45,46,47,48,49,50,51,52,53,54,55,56,57,58,59,60,61,62,63,64,65,66,67,68,69,70,71,72,73,74,75,76,77,78,79,80,81,82,83,84,85,86,87,88,89,90,91,92,93,94,95,96,97,98,99,100,101,102,103,104,105,106,107,108,109,110,111,112,113,114,115,116,117,118,119,120,121]. Forty-four of the 102 included studies were conducted with a cross-sectional study design, 35 as case–control studies, 14 as prospective cohort studies, 4 as randomized-controlled studies, 2 as retrospective cohort studies, 1 as cohort study, 1 as open label trial, and 1 as community survey. Fourteen studies (13.7%) were adult population-based, and 74 (72.5%) were adult population hospital-based. Regarding represented country, the most frequent one was Italy (n = 25; 24.5%), followed by USA (n = 23; 22.5%), India (n = 9; 8.8%), Brazil and Turkey (each n = 7; 6.8%), Iran (n = 5; 4.9%), Germany (n = 4; 3.9%), Switzerland (n = 3; 2.9%), Australia, Canada, France, Israel, Netherlands and Singapore (each n = 2; 2%), and New Zealand, Pakistan, Qatar, South Korea, Sweden, Taiwan and Venezuela (each n = 1, 1%). Two studies (2%) involving children/adolescents patients were population-based, while 12 (11.8%) were hospital-based. The total sample was made up of 24,035 patients (mean 239; min 15; max 2040). In all included studies diagnosis were made based on the application of ICD, DSM, or MINI diagnostic criteria and/or the use of validated assessment tools.

### 3.2. Comorbidity Rates

Comorbidities rates are reported in Table 2 and Table 3. Globally, 81 studies estimated the comorbidity rate of BD-OCD comorbidity. In detail, 44 assessed the lifetime prevalence of comorbid OCD in patients primarily affected by BD (Table 2), 31 assessed the lifetime prevalence of comorbid BD in patients primarily affected by OCD (Table 3), and 6 studies evaluated both prevalence rates.

#### 3.2.1. Comorbid OCD in BD Patients

Among population-based studies the lifetime prevalence of OCD comorbidity in BD subjects ranged between a minimum of 0.26% (n = 76) [24] and a maximum of 27.8% (n = 105) [32], with only one study differentiating BD-I (25.2%) and BD-II (20.8%) frequency in BD individuals [30]. With respect to hospital-based studies in adult populations, we identified a huge comorbidity frequency variation, with lifetime prevalence of comorbid OCD diagnosis in patients affected by BD ranged between 1.8% (n = 55) [55] and 100% in four studies (n = 56; n = 58; n = 64; n = 79) [66,87,88,89], although 6 studies out of 41 did not report the prevalence data [44,52,85,86,98,104]. Restricting the analysis to the eleven studies with a sample size greater than 250 individuals [36,37,58,62,64,65,73,78,91,93,95], the comorbidity prevalence range was slightly lower (3–23.2%) with a mean of 379.1 participants. Finally, when considering hospital-based studies including children and adolescents populations the prevalence data ranged between 2.4% (n = 85) [117] and 46.9% (n = 115) [108], with two other studies which achieved 20.7% (n = 82) [109] and 24.7% (n = 93) [119] respectively.

#### 3.2.2. Comorbid BD in OCD Patients

Nine population-based studies explored the lifetime prevalence of BD comorbidity in OCD subjects, with a huge range of findings (0.3–53.3%) [15,21,22,24,27,28,29,31,122]. However, only on study differentiated BD type (BD-II 30%) [15], and another one included both adult (n = 1078) and children and adolescent (198) populations, specifying two different prevalence data (6% and 5%) respectively [28].

### 3.3. Phenomenology

Thirty studies were dedicated to examining the characteristics of individuals experiencing comorbidity between BD and OCD, as summarized in Table 4. To assess obsessions and compulsions in these patients, the Yale-Brown Obsessive-Compulsive Scale (Y-BOCS) was predominantly employed, with the exception of one which used also the Dimensional Yale-Brown Obsessive-Compulsive Scale (DY-BOCS) [54], and another study that utilized a semi-structured in-depth interview to gather a wide range of data, including demographics, family history, psychopathological features, illness course, and symptoms [81,82,84]. Interestingly, the total Y-BOCS score did not conclusively demonstrate that BD-OCD patients exhibit more severe OCD symptoms compared to non-BD-OCD patients [100,105]. However, the Y-BOCS-11 sub-scale was linked to reduced insight into obsessive-compulsive symptoms among patients with comorbidity [74,76]. Notably, in individuals with BD, the co-occurrence of OCD was more likely to manifest during mixed manic for a few studies [64,77], mixed state in one case [101], and depressive episodes for many others [41,51,62,101]. Several types of obsessions were found to be positively associated with BD-OCD comorbidity, including sexual [75,81,82,92], symmetry [57,59,102], aggressive [59,92], religious [81,92], contamination [59,62,64,92], forbidden thoughts [40], saving [110], and hoarding obsessions [57,75,113,115,116]. Conversely, three studies reported lower rates of somatic and contamination obsessions and pathological doubt in patients with comorbidity compared to those without [74,92,102]. And one reported no specific effect on the phenomenology of OC symptoms [80]. Regarding compulsions, individuals with comorbidity were noted to exhibit higher rates of order [41,57,84,102,105,111], control/checking [59,62,81], repeating rituals [57,75,105], pathological slowness [74], reassurance seeking [74], cleaning [59], and counting [57] compulsions. In contrast, only three studies indicated lower rates of control, washing, ordering, and repeating compulsions in subjects with comorbidity [81,92,105]. In the presence of BD, children and adolescents with OCD were more frequently associated with hoarding obsessions and compulsions, as well as ordering compulsions [71,110,111,113,115,116]. Still, one study found that BD-OCD patients had significantly lower incidence of psychotic symptoms compared to BD group [93]. Only one study evaluated neuropsychological domains differences between patients with BD-OCD comorbidity compared to single disorders, concluding that comorbidity does not further impair the cognitive function [49,123].

### 3.4. Course of Illness

Table 5 provides a summary of the results from the included studies, highlighting the course of illness in both patients with and without a BD-OCD comorbidity.

#### 3.4.1. Age and Type of Onset

The data relating to which disorder precedes the other in comorbidity and at what age the diagnosis occurs vary greatly depending on the studies. Indeed, several studies have noted that OCD typically manifested simultaneously with the initial mood episode, rather than occurring before or after it [63,75,84,90,93,116,124], although there are also more recent findings reporting the identification of BD following an initial diagnosis of OCD [24,33,62,105,117], while a study found age of onset of BD, compared to the one of OCD, to be earlier in BD-OCD [80], and an early onset of BD and an early onset of depressive episodes were also associated with comorbid OCD [79]. More in detail, an ongoing multicenter 6-year naturalistic cohort study to examine the course of OCD in Netherlands concluded that patients with an early age of OCD onset presented higher rates of lifetime BD co-morbidity [33]. Conversely, a large study conducted in South Korea found that patients suffering from BD-OCD comorbidity presented earlier age-at-onset symptoms compared to BD alone. Moreover, in the majority of comorbid cases OCD onset preceded BD, followed by BD preceded OCD, and ultimately OCD began during the first mood episode [62]. In another investigation, it was found that individuals with both OCD and BD were more inclined to experience a gradual onset and episodic course of OCD symptoms when compared to individuals with OCD alone [81]. Indeed, the comorbidity group presented a slow and progressive development of OCD symptoms over an extended period, with initially mild, occasional obsessions or compulsions that gradually become more frequent and intense, compared to the acute or sub-acute onset of the pure OCD group.

#### 3.4.2. Course of Illness

Twenty-three studies were conducted to examine the characterization and progression of illness, some of them also categorizing patients into chronic and episodic subgroups (as indicated in Table 5). Several studies revealed that individuals with comorbid BD and OCD exhibited a higher prevalence of episodic OCD course when compared to patients without any comorbidity [74,80,81,82,84,90,102,103,105], with some studies reporting episodic OCD rates reaching as high as 75% in one particular study [105]. Notably, an Italian study found that episodic OCD was more frequently associated with BD-II comorbidity compared to chronic OCD [82]. However, it is noteworthy that the sole study that identified a more severe and persistent course of OCD in patients with both OCD and BD was conducted within a small cohort consisting mostly of 15 women, primarily diagnosed with BD-I [59], while a few studies did not identify any difference in illness duration [49,62,93]. Additionally, four separate studies involving BD patients revealed a higher incidence of a chronic or longer course of BD in those with comorbid OCD in comparison to patients without this comorbidity [36,60,64,68]. Furthermore, in various studies, the majority of BD-OCD subjects either experienced OCD exclusively during their depressive episodes or reported worsening of their OCD symptoms during periods of depression [90,105], with a study reporting improvement or remission during mania/hypomania phases [90]. In one report, it was demonstrated that 44% of BD-OCD patients experienced cycles where BD and OCD symptoms were intertwined [97]. When examined, it was observed that the total number of depressive episodes was greater in patients with the comorbidity of OCD-BD as opposed to those with BD alone [56,60], with one study identifying both more depressive and manic episodes [63].

#### 3.4.3. Global Functioning and Quality of Life

As indicated in Table 5, research conducted with both adult and pediatric patients consistently found that the coexistence of BD and OCD was linked to diminished functioning and a lower quality of life across all domains, including physical, psychological, social, educational, working and environmental aspects, in comparison to individuals with either ‘pure’ BD or ‘pure’ OCD [16,23,27,36,63,64,73,93,95,100,110,111,113]. Two hospital-based studies also observed similar trends, although they did not reach statistical significance [45,74].

#### 3.4.4. Suicide Ideation and Attempts

Within the Epidemiologic Catchment Area (ECA) database, and then confirmed by literature data [6], it was established that individuals with both OCD and BD exhibited statistically significant higher lifetime rates of various concerning factors such as ‘thoughts of suicide,’ ‘suicide attempts,’ ‘thoughts of death,’ and ‘wanting to die’ in comparison to those with BD alone [25]. Then, these findings have been further and consistently corroborated in recent studies involving OCD-BD patients [27,54,56,63,70,73,80,95] as well as in patients with BD-OC syndromes [121], even for OCD single diagnosis [90]. Additionally, among adolescents with BD, the presence of OCD was associated with a 2.4-fold increase in the likelihood of experiencing suicidal ideation when compared to adolescents affected by BD without this comorbidity [108]. However, it is noteworthy that a few studies did not find significant differences comparing lifetime suicide attempts rates between patients affected by BD with or without comorbid OCD [50,62,68]. In particular, a smaller study with a limited sample size (n = 35) did not find a statistically significant difference in terms of suicide attempts between individuals with ‘pure’ BD and those with OCD-BD [68]. Nevertheless, a larger study concluded that, in the BD-OCD group, more patients per-formed suicide attempts with violent methods, with a correlation between comorbid OCD and male gender and violent suicide attempts [50].

#### 3.4.5. Hospitalization

Nine studies explored hospitalization rate in BD-OCD comorbidity. Seven of them documented that patients with both BD and OCD exhibit elevated rates of hospitalization when compared to individuals without this comorbidity [45,63,67,74,100,110,113]. Differently, two hospital-based studies, one with a cohort study design [36] and one with a retrospective cohort design [93], did not identify any statistically significant difference in the number of hospitalizations between BD-OCD group and the BD one.

#### 3.4.6. Substance and Alcohol Abuse

Eight studies delved into the prevalence of substance abuse among individuals with comorbid BD and OCD, as presented in Table 5. A substantial hospital-based study involving a sizable cohort (n > 500) revealed that BD-OCD patients had more than double the likelihood of being diagnosed with substance use disorders (odds ratio [OR] 3.18, 95% confidence interval [CI] = 1.81–5.58) and alcohol use disorders (OR 2.21, 95% CI = 1.34–3.65) when compared to those without this comorbidity [100]. These results were consistent with both earlier [16,39,73,75] and following research findings [27,100]. Additionally, one study demonstrated a positive correlation between BD-OCD comorbidity and the abuse of sedatives, nicotine, alcohol, and coffee [84]. The ECA study further validated the heightened rate of drug abuse among individuals with OCD-BD comorbidity in comparison to those without comorbidity, with rates of 37.1% versus 28.2%, respectively [25].

#### 3.4.7. Other Psychiatric and General Medicine Comorbidities

Ten investigations examined the co-occurrence of other psychiatric and general medicine diseases among individuals with the BD-OCD comorbidity, as presented in Table 5. Consistent with both prior [60,84] and subsequent [62] research, one study demonstrated that individuals with BD and OCD were more than twice as likely to receive diagnoses of panic disorder, agoraphobia, post-traumatic stress disorder, and eating disorders compared to those without this comorbidity [100]. These findings were also confirmed by more recent research that found that the BD-OCD group presented higher rates of panic disorders with agoraphobia and impulse control disorders compared to patients affected by OCD alone [54]. In evaluations conducted with children and adolescents, it was observed that BD-OCD patients exhibited higher rates of attention-deficit/hyperactivity disorder and oppositional defiant disorder, along with lower rates of generalized anxiety disorder when compared to patients without this comorbidity [111,113]. Additionally, although not statistically significant, BD-OCD comorbidity has been found to be more common in patients affected also by adult separation anxiety disorder [99]. Only two studies investigated comorbid personality disorder. One of them has been carried out in Italy, and investigated personality disorders comorbidities comparing individuals with BD and OCD to those without this comorbidity; this study reported increased rates of at least one Cluster A personality disorder, at least one Cluster B personality disorder, as well as narcissistic and antisocial personality disorders in individuals with this comorbidity [75]. In the second one, BD-OCD patients presented higher rates of comorbid social anxiety and anxious avoidant personality disorder [93]. Regarding other general medical comorbidities, only two studies were identified, with partial opposite results. In fact, in the first of these, BD-OCD patients presented higher rate of medical comorbidity compared to BD patients without OCD. In this case the medical burden was assessed according to the Cumulative Illness Rating Scale (CIRS) with the most common medical conditions including obesity, migraines, hypertension, hyperlipidemia, and asthma [65]. While in the second, BD-I-OCD patients presented fewer overall medical comorbidities, collected by medical records, compared to BD-I patients, but a higher frequency of cardiovascular conditions [64].

#### 3.4.8. Heredity

Only one study investigating the familial transmission of comorbid BD-OCD was identified (Table 4) [63]. Indeed, in a sample of 32 patients affected by BD-OCD, authors found that first- and second-degree relatives had higher rates of BD-OCD and OCD, but not of BD [63]. Instead, eleven studies, comprising eight hospital-based and three population-based investigation, examined family history of either OCD or BD in individuals with comorbid BD-OCD using semi-structured or unstructured clinical interviews and clinical records [16,24,36,54,64,68,74,80,84,93,105]. Among these, five studies revealed that BD-OCD patients exhibited a higher prevalence of family history for mood disorders [36], and some of them were also associated with a lower prevalence of family history for OCD compared to individuals with non-BD-OCD [54,74,84,105], while one study reported the opposite pattern in terms of OCD and none significant differences in family history for BD [68]. On the other hand, two studies found that in the BD-OCD population first- and second-degree relatives had higher rates of OCD rather than BD [63,93]. While another study identified higher frequency for both BD and OCD among relatives of individuals suffering from the comorbidity [64]. Finally in two studies, authors concluded without identifying any difference in family history of OCD and BD in patients affected by BD-OCD [15,80]. Additionally, concerning the course of illness, it was noted that a family history of mood disorders was more frequently reported in episodic OCD patients compared to those with a continuous or chronic course of OCD [82].

#### 3.4.9. Biological Markers

Within the scope of biological markers pertaining to comorbidity between BD and OCD, only one study was identified, and it is summarized in Table 4. Researchers based in the USA conducted an analysis of serotonin transporter (5-HTT) binding potential (BP) utilizing positron-emission tomography (PET) and [11C] DASB, a radioligand known for its high sensitivity and specificity in targeting 5-HTT. They observed that individuals with comorbid BD-OCD displayed higher 5-HTT-binding potential than those with BD alone in several brain regions, including the insula, posterior cingulate cortex, subgenual anterior cingulate cortex, and dorsal cingulate cortex [43].

#### 3.4.10. Treatment

There have been only four double-blind randomized clinical trials (RCTs) conducted in individuals with both BD and OCD comorbidity. On the other hand, fifteen other studies, with twelve including adults and three including child/adolescent populations, examined non-randomized treatment responses identified in patients (as summarized in Table 4). In the first 16-week RCT 58 patients in the manic phase of BD who had OC symptoms were randomly allocated to receive memantine or placebo plus their routine medications (lithium + olanzapine + clonazepam). Authors concluded that memantine, as an adjunctive agent, was more effective than placebo in decreasing the OC symptoms (more than 34% decrease in mean YBOCS score) during manic phase [87]. In the second RCT authors aimed to evaluate the effects of aripiprazole as an adjuvant treatment (to lithium + clonazepam) for OC symptoms in patients with BD-I, manic phase. According to their results, aripiprazole showed effectiveness in reducing OC symptoms (more than 34% decline in YBOCS score) in BD-OCD patients compared to placebo (91.30% vs. 4.34%) [88]. The third double-blind RCT examined the efficacy of quetiapine as an adjuvant treatment for OC symptoms in a population of 47 patients with BD-I in euthymic phase randomly allocated to receive either quetiapine or placebo plus their routine medications (lithium + clonazepam) [89]. Authors found that quetiapine showed greater effectiveness than placebo in reducing OC symptoms (more than 34% decline in Y-BOCS score (*p* < 0.001)) in BD-OCD pt. (Y-BOCS score from 24.37 to 15.26 (*p* < 0.001)) compared to placebo (Y-BOCS from 24.21 to 23.94 (*p* = 1.97)), without reporting any serious adverse effects in both groups [89]. Finally, the last double blind RCT included tried to explore if adjunctive therapy of aripiprazole or risperidone to valproate sodium may be an effective in the treatment of OC symptoms in a population of 64 BD-OCD patients [66]. According to these results, aripiprazole is more effective than risperidone in the treatment of OCD in BD-OCD patients, and higher dose and longer duration of treatment significantly increases the therapeutic effect of adjunctive therapy of these two drugs in the treatment of BD-OCD comorbidity [66]. The other studies selected did not identify a single treatment modality. Overall, most authors have used mood stabilizers [67,80,93,101], with lithium and valproate being used more frequently [35,84,90,113]. There has often been the addition of antipsychotics [35,63,64,80,84,101], mainly second generation and more frequently olanzapine [67,84,113]. The role of olanzapine is particularly relevant in the BD-OCD comorbidity, since an analysis of data derived from three identically designed open-label trials focusing on olanzapine treatment in youth with BD, conducted in the USA, revealed that the treatment response in individuals with OCD-BD comorbidity was notably lower than that in patients without this comorbidity. Interestingly, no statistically significant differences were observed in the rates of treatment discontinuation or adverse effects [109]. The data on antidepressants appear more confusing, as they are highly influenced by the phase of bipolar disorder, the risk of affective switch, and the severity of the anxious-obsessive symptoms [35,63,64,80,93,101]. Indeed, there is a study in which antidepressants were not used to treat obsessive symptoms in comorbidity, since drug-induced (hypo)manic switch was observed in more than 60% of the BD-OCD patients enrolled who were previously exposed to antidepressants [62]. In another research, antidepressants were not used during mood episodes [93], while in other works they were used widely regardless of the stage or the presence or absence of BD, in association with mood stabilizers [64,80]. In another of these studies, it was reported that among BD-OCD patients, the use of clomipramine, and to a lesser extent, selective serotonin reuptake inhibitors (SRIs), was associated with a higher occurrence of manic switches compared to patients without this comorbidity. Furthermore, BD-OCD patients who did not concurrently receive mood stabilizers exhibited a more frequent occurrence of pharmacological-induced mania compared to non-BD-OCD patients. In 55% of BD-OCD patients, a combination of mood stabilizers was deemed necessary, while 10.5% of them required a combination of mood stabilizers and atypical antipsychotics [84]. Finally, only two studies, one in adult [54] and one in child/adolescent [116] population, psychotherapy has been reported as effective therapy, more frequently then single disorders groups. Regarding child and adolescent population, another study indicated that young individuals with both BD and OCD more frequently received multiple medications rather than SRIs alone. Additionally, they were more likely to be prescribed mood stabilizers and exhibited higher rates of non-response to pharmacological treatment when compared to individuals with OCD who did not have BD [113,114]. Still, in a more recent study conducted with a child and adolescent population, BD-OCD group more frequently received psychotherapy and second generation antipsychotics compared to BD and OCD alone, and they presented the poorest outcome in terms of response to treatments [116].

## 4. Discussion

The co-occurrence of two major psychiatric disorders such as BD and OCD has long intrigued researchers and clinicians alike. Indeed, this comorbidity raises complex diagnostic and treatment challenges and prompts important questions about the nature of these two psychiatric conditions. Our study sheds light on several key aspects of this comorbidity, including epidemiology and type of onset, phenomenology, course of illness, clinical implications and functioning, suicide risk, hospitalizations, other overlapping comorbidities, and treatment management strategies. In accordance with Kraepelin’s perspective, the most reliable way to establish a psychiatric diagnosis is through the long-term course of the illness [125]. Therefore, it may be supposed from the existing evidence that the majority of cases initially labeled as ‘comorbid’ BD-OCD may be, in fact, instances of BD, with OC symptoms emerging as epiphenomena of BD during depressive or manic mood episodes [126], although this currently represents more of a hypothesis than evidence. The frequent prior occurrence of OC symptoms in childhood or early adolescence, before the first clear affective episode, could be interpreted as the manifestation of prodromes (heterotypic trajectory) of BD. The episodic course of OCD/OC symptoms when “comorbid” with BD (OCD as a stand-alone illness has in the vast majority of cases a chronic course), with fluctuations in the severity of OC symptoms that tend to cycle together with affective sympotms, further confirms our interpretation of the so-called comorbidity. As for other diagnostic criteria, the findings were varied, primarily suggesting heightened severity of the illness or improved functioning, but failing to provide definitive confirmation of the diagnosis. These conclusions have been drawn after a meticulous assessment of the included observational studies, as detailed in Table 1, taking into account factors such as research design, sample size, and statistical analysis. Our findings align with previous research, indicating a substantial overlap between BD and OCD [6]. The prevalence of comorbidity appears to be significant, with approximately 50–75% of OCD cases manifesting primarily during mood episodes, especially depressive ones, in BD. This suggests that the majority of comorbid OCD cases may be secondary to the mood disturbances seen in BD. However, a notable minority of cases exhibit OCD symptoms independently of BD. This highlights the need for careful assessment and differentiation in clinical practice, as the management of BD with comorbid OCD may differ from that of “pure” OCD cases [127]. Overall, OC symptoms tend to emerge more frequently, and sometimes exclusively, during depressive episodes in comorbid patients. Interestingly, comorbid BD and OCD can cycle together, with OC symptoms often improving during manic or hypomanic episodes. Additionally, there’s evidence indicating that serotonin reuptake inhibitor (SRI) antidepressants can trigger more instances of mania or hypomania (or mixed features) in individuals with BD and OCD compared to those without this comorbidity, or destabilize the course of BD (exactly as they do without proper mood stabilizers in individuals with BD). In some cases, the use of SSRIs or clomipramine (first line compounds in OCD) is even associated with a “paradoxical” increase in the severity of OC symptoms in patients with “comorbid” OCD and BD [128], while the addition of other mood stabilizers to lithium or valproate is beneficial also on OC symptoms. [89,129,130]. These findings have significant implications for the clinical management of patients dealing with both BD and OCD, suggesting that physicians should exercise caution when considering antidepressant treatment for comorbid OC symptoms in BD. In fact, such an approach might potentially exacerbate BD by inducing manic or hypomanic states through SRIs [131,132,133]. These findings may also be explained using the concept of a diagnostic hierarchy within the field of psychiatry, in contrast to the more egalitarian approach found in the DSM system [134]. In the hierarchical perspective, which is rooted in traditional European psychopathology, conditions like OCD with anxiety presentations are not identified as distinct disorders when they co-occur with mood presentations such as BD. To put it differently, OCD would only be diagnosed if BD had been ruled out. Whether this interpretation was accurate, as some results included in our review seem suggest, it implies that clinicians evaluating patients with OCD should also consider examining family histories for mood disorders and other indicators of bipolar tendencies. If the majority of OCD symptoms are epiphenomena of BD, it is plausible that both sets of symptoms could potentially respond positively to adequate mood stabilizer treatment. In cases where a minority of individuals experience persistent OCD symptoms despite improvement in mood episodes, the option of adding low doses of antidepressants may be considered, provided that strict monitoring for emerging manic or mixed states is maintained. Notably, several case reports have documented the amelioration of OC symptoms in comorbid patients through the use of mood stabilizers [128,135] and atypical antipsychotics [136]. The insights from this review underscore the need for more comprehensive treatment research involving these agents alone, without the concurrent use of serotonin reuptake inhibitors (SRIs), in individuals with comorbid BD and OCD. An argument can be made that a higher occurrence of depressive episodes alongside OCD could be a consequence of inadequate response to OCD treatment. Data from the STEP-BD study [137] indicated that the presence of anxiety disorders, including OCD, was associated with an elevated risk of relapse into depression. However, this argument is flawed in two significant ways. Firstly, it fails to explain the presence of OCD during manic episodes, a phenomenon that doesn’t align with a treatment-related theory. Secondly, it relies on a somewhat improbable assumption: it would imply that every time an individual experiences a depressive episode, it is caused by an exacerbation of OCD, and this co-occurrence coincidentally repeats dozens of times over a lifetime, especially considering that individuals with mood disorders (bipolar and unipolar) tend to experience numerous episodes over their lifetime. While this is theoretically possible, it remains unexplored and unproven. On the contrary, the alternative perspective, rooted in over a century of robust scientific research [125], suggests that mood disorders involve numerous mood episodes throughout a person’s lifetime. The observation of OCD symptoms occurring concurrently with some of these numerous mood episodes is more plausibly linked to the ongoing mood disorder itself rather than intermittent OCD symptoms being the primary cause. However, it is essential to acknowledge that the question of causation cannot be definitively settled based solely on clinical data, whether retrospective or prospective. Correlation and causation are distinct concepts, as widely recognized, and a comprehensive examination of other facets of causation, as advocated by the renowned epidemiologist A. Bradford Hill [138], is necessary to provide a more complete understanding of this complex relationship. Regarding the concept of comorbidity, we adhere to Feinstein’s definition, which defines it as the presence of a “distinct additional clinical entity” [1]. In Feinstein’s formulation, the implication was that an entirely different and independent disease occurs concurrently with another disease, often in a seemingly random manner. In contrast, the Diagnostic and Statistical Manual of Mental Disorders (DSM) explicitly outlines overlapping clinical criteria for many diagnoses, particularly within mood and anxiety disorders, thus guaranteeing comorbidity in a different sense than the medical definition, where it denotes the co-occurrence of independent diseases. Using the DSM’s definition, it becomes unclear whether simultaneous diagnoses truly signify the presence of distinct clinical entities or rather signify multiple expressions of a single clinical entity [3,139]. Following Feinstein’s definition, “true” comorbidity between OCD and BD would imply the coincidental co-occurrence of two independent diseases. This would be estimated by multiplying their individual prevalence rates, which are approximately 1% for each condition, resulting in an expected true comorbidity rate of just 0.1% in the general population. In light of the results of this systematic review, we can conclude that the comorbidity between BD and OCD is a complex and multifaceted phenomenon with peculiar clinical, diagnostic, and treatment implications. Our study contributes to the growing body of literature in this area and emphasizes the need for a nuanced and evidence-based approach to managing individuals with this comorbidity. Further research is warranted to unravel the underlying mechanisms, improve diagnostic accuracy, and refine treatment strategies to enhance the well-being of patients affected by BD-OCD comorbidity.

### Limitations and Strengths

Upon interpreting the results presented in this systematic review, we recognize some limitations and strengths that should be considered. Firstly, a majority of the included studies adopt a cross-sectional observational design, featuring retrospective data collection and frequently lacking a control group, as indicated by the employed quality assessment scale. This significantly hampers the generalizability of the results, given that a prospective and early assessment of comorbidity development, although clinically challenging, would be highly beneficial. Moreover, the epidemiological estimate of comorbidity is also strongly influenced by the different designs of the studies, which, not being only population studies, also reaches peaks of 100% comorbidity, which cannot be used as reference data in the psychiatric population. Secondly, a significant portion of the studies was conducted in outpatient clinics, enrolling patients who are clinically stable, in remission, in a hypomanic phase, or in a non-severe depressive phase, often with small sample sizes. The scarcity of hospital-based studies with participants in manic or severe depressive phases constrains the ability to draw conclusions regarding clinical severity and prognosis. Additionally, a limited number of studies conducted in hospital settings examine patients during clinical remission, with a higher frequency of research focusing on BD subjects during depressive episodes rather than manic ones. Notably, these studies may be susceptible to potential confounding variables, including demographic and historical illness factors, which are often inadequately addressed through multivariate modeling. Consequently, these factors collectively limit the generalizability of the findings. On the other hand, we highlight the inclusion of both studies with a primary diagnosis of BD and with a primary diagnosis of OCD, which allowed us to speculate on possible differences in the clinical presentation based on the leading disorder. Furthermore, we established strict inclusion standards that required compliance with DSM diagnostic criteria established by trained investigators using validated assessment scales, mainly with high interrater reliability, in order to only include studies with recognized diagnoses of both single and comorbid BD and OCD, avoiding attenuated, subthreshold, or spectrum forms. Moreover, another strength of this review lies in its systematic approach, encompassing the entirety of the scientific evidence available to date across major medical databases. However, it is imperative to underscore the necessity for further research to either validate or challenge our findings and the subsequent clinical recommendations. Specifically, studies delving into hereditary and biological markers are essential to shed light on the pathogenic mechanisms underpinning the co-occurrence of BD and OCD. Such research will also help clarify the diagnostic validity of this comorbidity and inform the development of more effective treatment approaches.

## 5. Conclusions

BD-OCD comorbidity represents a clinical challenge and a research frontier that needs to be further investigated to better characterize the role played by heredity and the potential application of biomarkers to strengthen its diagnostic validity and identify the most effective management approach. The evidence collected in this review traces the profile of BD-OCD comorbidity as a peculiar form of disorder. Moreover, our findings allow us to speculate that the OCD may be an epiphenomenon of comorbid BD, suggesting that this comorbidity likely has various underlying causes and clinical manifestations, with phenotypic and psychopathological characteristics that differentiate it from both BD and OCD alone, and this cannot be explained by the mere overlap of two conditions.

## Figures and Tables

**Table 1:** Studies that met inclusion/exclusion criteria for systematic review.

References	Study Design	Country	Study Population	Sample Size	Diagnosis Assessment	Outcomes	Quality *
Population-based studies: adults							
Abramovitch et al., 2019 [21]	Prospective cohort study	Netherlands	2.067 subjects recruited (mean age 36.86 ± 11.05): OCD (n = 316)	316	SCID-I, Y-BOCS; DSM-IV	PR	26/31
Adam et al., 2012 [22]	Cross-sectional study	Germany	4181 subjects (age range = 18–65): OCD (n = 30)	30	Broad concept of OCD, DIA-X/M-CIDI; DSM-IV	PR	25/31
Angst et al., 2004 ** [15]	Prospective cohort study	Switzerland	591 subjects recruited at age 19/20 and assessed over 20 years: OCD (n = 30)	30	Broad definition for BD and OCD; DSM-IV	PR	26/31
Angst et al., 2005 ** [16]	Prospective cohort study	Switzerland	591 subjects recruited at age 19/20 and assessed over 20 years: OCD (n = 30), BD (n = 93)	123	Broad definition for BD and OCD; DSM-IV	HE, CI	26/31
Carta et al., 2020 [23]	Community survey	Italy	2.267 subjects recruited (age > 18)	44	ANTAS-SCID, SF-12, MDQ; DSM-IV	PR	22/31
Cederlöf et al., 2014 [24]	Prospective cohort study	Sweden	OCD (n = 19.814) and BD (n = 48.180) patients included in the Swedish Patient Register between January 1969 and December 2009: BD-OCD (n = 76), OCD-BD (n = 787)	76/787	NS; ICD-10	CI, HE, PR, TR	23/31
Chen et al., 1995 [25]	Cross-sectional study	USA	Pt. with BD, unipolar disorder or any Axis I disorder other than bipolar or unipolar disorder (n = 6622, age > 18): BD (n = 167)	167	DIS; DSM-III	PR, CI	25/31
Faravelli et al., 2004 [26]	Cross-sectional study	Italy	2500 subjects randomly selected from the lists of 15 GPs: BD (n = 19, age > 14), OCD (n = 57, age > 14)	76	SCID; DSM-IV	PR	24/31
Fineberg et al., 2013 [27]	Prospective cohort study	Switzerland	591 subjects recruited at age 19/20 and assessed over 30 years: OCD (n = 30)	30	SCL-90; CGI; DSM-IV	PR, PH, CI	23/31
Fireman et al., 2001 [28]	Cross-sectional study	USA	1.728.480 subjects (age > 6): adults with OCD (n = 1078), children and adolescents with OCD (198)	1078/198	NS; DSM-IV	PR	24/31
Huang et al., 2014 [29]	Prospective cohort study	Taiwan	OCD patients, age > 18, with comorbid diagnosis: 1763; OCD patients, age ≤ 18, with comorbid diagnosis: 277	1763/277	NS; ICD-9-CM	PR	23/31
Merikangas et al., 2007 [30]	Cross-sectional study	USA	9282 subjects recruited (age ≥ 18): adults with any BD (n = 408)	408	CIDI, SCID; DSM-IV	PR	25/31
Subramaniam et al., 2020 [31]	Cross-sectional study	Singapore	6126 recruited subjects (4258 completed the study): OCD (n = 217, age ≥ 18)	217	WHO-CIDI, YMRS, QIDS-SR, SDS, SF-12, MCS, PCS, MSPSS; DSM-IV	PR	24/31
Teh et al., 2020 [32]	Cross-sectional study	Singapore	6126 recruited subjects (4258 completed the study): BD (n = 94, age ≥ 18)	94	WHO-CIDI version 3.0, YMRS, QIDS-SR, SDS; DSM-IV	PR	23/31
Hospital-based studies: adults							
Anholt et al., 2014 [33]	Cross-sectional study	Netherlands	419 recruited subjects (377 included in the study, mean age 36.3 ± 11.2)	377	SCID-I, Y-GTSS, BAI, BDI, Y-BOCS, AQ; DSM-IV	CI	23/31
Baptista et al., 2020 [34]	Cross-sectional study	Venezuela	BD (n = 40, mean age 46.6 ± 13.5), OCD (n = 42, mean age 35.2 ± 13.5)	82	Y-BOCS, YMRS, MDQ; DSM-IV	PR	22/31
Benatti et al., 2014 [35]	Cross-sectional study	Italy	OCD (n = 75, mean age 41.62 ± 10.78)	75	SCID-I, -II, BIS, Y-BOCS; DSM-IV	PR, PH, TR	22/31
Bener et al., 2016 [36]	Cohort study	Qatar	BD (n = 396, mean age 41.4 ± 12.28)	396	SCID, WMH-CIDI, Y-BOCS, HAM-D, YMRS; DSM-IV	PR, PH, CI, HE	21/31
Berkol et al., 2021 [37]	Retrospective cohort study	Turkey	BD (n = 255, mean age 40.7 ± 12.6), of which BD-I (n = 244), BD-II (n = 11)	255	SCID-I/CV; DSM-IV	CI, HE, PH, PR, TR	20/31
Bogetto et al., 1999 [38]	Case control study	Italy	OCD (n = 160, mean age: males 32.1 ± 13.0, females 36.9 ± 11.4)	160	NS; DSM-IV	PR	21/31
Boylan et al., 2004 [39]	Prospective cohort study	Canada	BD (n = 138, age range = 16–65)	138	SCID; DSM-IV	PR	22/31
Bramante et al., 2021 [40]	Cross-sectional study	Italy	OCD (n = 601, mean age 35.0 ± 12.5	601	SCID-I, Y-BOCS, HAM-A, HAM-D, SCID-PQ; DSM-5	PR, PH	22/31
Braverman et al., 2021a ** [41]	Cross-sectional study	Israel	BD (n = 73, mean age 32.9 ± 10.4): BD without OCD (n = 37, mean age 33.1 ± 10.4), BD-OCD (n = 19, mean age 3.6 ± 10.4), BD-OCD subthreshold (n = 17, mean age 33.1 ± 10.6)	73	SCID I/P, HAM-D, Y-BOCS, YMRS; DSM-5	PR, PH	21/31
Braverman et al., 2021b ** [42]	Cross-sectional study	Israel	BD (n = 70): BD-OCD (n = 27, mean age 33.3 ± 10.3), BD (n = 43, mean age 33.5 ± 10.7)	70	SCID I/P, HAM-D, Y-BOCS, YMRS, DSM-5	PR, PH	21/31
Cannon et al., 2006 [43]	Case control study	USA	BD (n = 18, mean age = 30 ± 9)	18	NS; DSM-IV	BM	23/31
Cassano et al., 1999 [44]	Cross-sectional study	Italy	Pt. with psychotic symptoms consecutively hospitalized (n = 77, mean age = 33.5 ± 10.3): BD-I (n = 48), schizoaffective disorder, bipolar type (n = 11), unipolar depression (n = 18)	48	SCID-P; DSM-III-R	PR	23/31
Centorrino et al., 2006 [45]	Case control study	USA	Adults (n = 62) with BD, OCD, or BD-OCD	62	NS; DSM-IV	CI	20/31
Cosoff et al., 1998 [46]	Case control study	Australia	Subjects with a psychotic disorder (n = 100, mean age: men = 34.8 ± 10.0, women = 34.9 ± 9.6): BD (n = 20)	20	SCID-P; DSM-III-R	PR	20/31
Craig et al., 2002 [47]	Cross-sectional study	USA	450 subjects: BD with psychosis (n = 138, mean age range = 15–60)	138	SCID; DSM-III-R	PR	22/31
Das et al., 2013 [48]	Prospective cohort study	India	BD-I mania (n = 84, age 30.85 ± 8.76)	84	YMRS, Brief Psychiatric Rating Scale, HAM-A, SADS-L; DSM-IV-TR	PR	26/31
de Filippis et al., 2018 [49]	Cross-sectional study	Italy	68 recruited subjects: BD (n = 22, mean age 47.9 ± 11.8), BD-OCD (n = 26, 47.38 ± 13.2), 20 OCD (42.5 ± 15.5)	68	MINI, YMRS, HAM-D, MMSE, Y-BOCS, CGI, RCFT, IGT, WCST, TMT, HSct; DSM-IV-TR	PR, PH, TR	20/31
Di Salvo et al., 2020 [50]	Cross-sectional study	Italy	BD (n = 990, mean age 49.0 ± 15.6): BD without OCD (n = 789, mean age 50.7 ± 15.5), BD-OCD (n = 201, mean age 42.3 ± 14.1)	990	DSM-IV-TR, DSM-5	PR, CI	22/31
Dell’Osso et al., 2000 [51]	Case control study	Italy	BD with psychotic features (n = 125, mean age: 33.3 ± 11.1)	125	SCID-P; DSM-III-R	PH	21/31
Dilsaver et al., 2008 [52]	Case control study	USA	187 Latino pt. enrolled consecutively from 2001 to 2003: BD-I (n = 69, mean age = 34.9 ± 11.8)	69	SCID-CV; DSM-IV	PR	20/31
Diniz et al., 2004 [53]	Cross-sectional study	Brazil	OCD (n = 161, mean age = 30 ± 10))	161	SCID; DSM-IV	PR	21/31
Domingues-Castro et al., 2019 [54]	Cross-sectional study	Brazil	OCD (n = 955, mean age 35.8 ± 12.5): OCD without BD (n = 881, mean age 35.9 ± 12.6), OCD-BD (n = 74, mean age 34.9 ± 10.7	955	SCID-I, Y-BOCS, DY-BOCS, OCD Natural History Questionnaire, BDI, BAI, BABS, USP Sensory Phenomena Scale, Suicidality Assessment; DSM-IV-TR	CI, HE, PH, PR, TR	22/31
Edmonds et al., 1998 [55]	Case control study	New Zealand	BD (n = 55, mean age = 41.6), first-degree relatives (n = 67, mean age = 50.3)	122	DIGS; DSM-IV	PR	20/31
Goes et al., 2012 [56]	Cross-sectional study	USA	BD (n = 1416, mean age = 42.0), first-degree relatives with BD (n = 850)	1416/850	DIGS; DSM-IV	CI	23/31
Hantouche et al., 2003 [17]	Case control study	France	OCD (n = 628, mean age CYC-OCD = 35 ± 12, mean age NC-OCD = 36 ± 14)	628	NS; DSM-IV	PR	24/31
Hasler et al., 2005 [57]	Cross-sectional study	USA	OCD (n = 317, age > 18)	317	SCID; DSM-IV	PH	23/31
Henry et al., 2003 [58]	Cross-sectional study	France	BD (n = 318, mean age = 53.3 ± 15.1)	318	DIGS; DSM-IV	PR	23/31
Issler et al., 2005 [59]	Case control study	Brazil	OCD-BD (n = 15, mean age = 38.9 ± 10.7)	15	SCID-P; DSM-IV	PH, CI	14/31
Issler et al., 2010 [60]	Case control study	Brazil	BD (n = 30, mean age: BD = 41.8 ± 10.5, OCD-BD = 38.9 ± 10.7)	30	SCID-P; DSM-IV	CI	17/31
Jakubovski et al., 2013 [61]	Prospective cohort study	Brazil	OCD (n = 196, mean age = 33.6 ± 10.6)	196	SCID-I, Y-BOCS DY-BOCS, BDI, BAI; DSM-IV-TR	PR	26/31
Jeon et al., 2017 [62]	Cross-sectional study	South Korea	BD (n = 314, mean age = 34.9 ± 11.6)	314	SCID, Y-BOCS, OCI-R-K	PR, CI, PH, TR	21/31
Kazhungil et al., 2017 [63]	Cross-sectional study	India	BD (n = 90): BD-OCD (n = 20, mean age = 41.03 ± 11.77), BD with obsessive–compulsive symptoms (n = 32, mean age = 39.36 ± 11.24)	90	Structured Clinical Interview for DSM-IV Axis I Disorders, GAF, HDRS, YMRS, Y-BOCS, FIGS	PR, CI, HE, PH	20/31
Khan et al., 2019 [64]	Cross-sectional study	Pakistan	BD-I (n = 469)	469	DSM-IV-TR	PR, CI, HE, PH, TR	21/31
Kemp et al., 2014 [65]	Cross-sectional study (LiTMUS)	USA	BD (n = 264, mean age = 39.2 ± 12.4)	264	SCID, MADRS, YMRS, QIDS-SR, the Quality of Life, Enjoyment, Q-LES-Q, LIFE-RIFT, CIRS; DSM-IV	PR, CI	20/31
Khorshidian et al., 2023 [66]	RCT	Iran	64 pt. recruited; 61 completed the study (31 in the risperidone group and 30 in the aripiprazole group; mean age was 38.9 in the risperidone group and 40.1 in the aripiprazole group)	64	DSM-5, HDRS, YMRS, Y-BOCS	PR, TR	28/31
Kim et al., 2014 [67]	Prospective cohort study	Australia	BD (n = 174): BD-OCD (n = 24, mean age = 39.8 ± 12.0)	174	MINI, YMRS, HAM-D; DSM-IV	PR, CI, TR	26/31
Koyuncu et al., 2010 [68]	Case control study	Turkey	BD (n = 214, mean age: BD = 34.8 ± 10.3, OCD-BD = 36.2 ± 15.9)	214	SCID; DSM-IV	PR, HE, CI	20/31
Kruger et al., 1995 [69]	Cross-sectional study	Germany	Major affective disorder (n = 149, mean age = 49 ± 12): BD (n = 44)	37	DIS; DSM-III	PR	21/31
Kruger et al., 2000 [70]	Case control study	Germany	BD-I or BD-II (n = 143, mean age = 44)	143	SCID; DSM-III-R	PR, CI	22/31
LaSalle-Ricci et al., 2006 [71]	Cross-sectional study	USA	OCD (n = 204, age > 18)	204	SCID-P for DSM-IV	PH	20/31
Lensi et al., 1996 [72]	Case control study	Italy	OCD (n = 263, mean age = 33.1)	263	NS; DSM-III-R	PR	23/31
Magalhaes et al., 2010 [73]	Case control study	Brazil	BD (n = 259, mean age = 41)	259	SCID; DSM-IV	PR, CI	23/31
Mahasuar et al., 2011 [74]	Case control study	India	OCD (n = 91, mean age: OCD = 29.36 ± 8.31, BD-OCD = 28.39 ± 7.10)	91	SCID for DSM-IV	PH, HE, CI	19/31
Maina et al., 2007 [75]	Case control study	Italy	OCD (n = 204, mean age = 34.7 ± 12.1)	204	SCID; DSM-IV	PR, PH, CI	21/31
Marazziti et al., 2002 [76]	Cross-sectional study	Italy	OCD (n = 117, mean age = 30 ± 9.3)	117	SCID-P; DSM-IV	PR, PH	21/31
McElroy et al., 1995 [77]	Case control study	USA	BD (n = 71, mean age = 35 ± 16)	71	SCID-P; DSM-III-R	PH	23/31
McElroy et al., 2001 [78]	Case control study	USA	BD-I or BD-II (n = 288, mean age = 42.8 ± 11.3)	288	SCID-P; DSM-IV	PR	23/31
Ortiz et al., 2011 [79]	Cross-sectional study	Canada	BD (n = 379, mean age = 25.1 ± 10.6)	379	SADS-L; DSM-IV	CI	23/31
Ozdemiroglu et al., 2017 [80]	Cross-sectional study	Turkey	BD (n = 48, mean age = 42.3 ± 12.4), OCD (n = 61, mean age = 37.7 ± 12.9), BD-OCD (n = 32, mean age = 36.2 ± 12.0)	141	SCID-I, Y-BOCS, YMRS, BDI, BIS-11; DSM-IV	PR, CI, HE, PE, PH, TR	21/31
Perugi et al., 1997 [81]	Case control study	Italy	OCD (n = 315, mean age: BD-OCD = 32.8 ± 12.2, OCD = 32.5 ± 12.6)	315	NS; DSM-III-R	PR, PH, CI	22/31
Perugi et al., 1998 [82]	Case control study	Italy	OCD (n = 135, mean age = 38.4 ± 13.3)	135	NS; DSM-III-R	PR, HE, CI	21/31
Perugi et al., 1999 [83]	Case control study	Italy	269 pt. enrolled consecutively from 1993 to 1995: OCD (n = 79, mean age = 30.4 ± 11.8)	79	SCID-Up-R; DSM-III-R	PR, TR	20/31
Perugi et al., 2002 [84]	Case control study	Italy	OCD-MDE (n = 68, mean age = 34.2 ± 12.5); BD-OCD (n = 38, mean age = 35.9 ± 12.2)	68	SCID; DSM-IV	PR, PH, CI, HE, TR	20/31
Pini et al., 1997 [85]	Case control study	Italy	Current episode of depression (n = 87): bipolar depression (n = 24, mean age = 37.9 ± 12.0), unipolar depression (n = 38, mean age = 47.0 ± 15.0), dysthymia (n = 25, mean age = 43.0 ± 12.0)	24	SCID-P; DSM-III-R	PR	20/31
Pini et al., 1999 [86]	Cross-sectional study	Italy	BD (n = 125, age > 16)	125	SCID-P; DSM-III-R	PR	21/31
Sahraian et al., 2017 [87]	RCT	Iran	58 BD pt. recruited; 38 completed the study (19 in the memantine group and 19 in the placebo group; mean age 34.21 ± 10.18 and 32.26 ± 10.30, respectively)	58	Y-BOCS; DSM-IV-TR	PR, TR	28/31
Sahraian et al., 2018 [88]	RCT	Iran	56 BD pt. recruited; 46 completed the study (23 in aripiprazole group and 23 in placebo group; mean age 34.10 ± 11.60 and 39.4 ± 14.20, respectively)	56	Y-BOCS, YMRS; DSM-IV	PR, TR	28/31
Sahraian et al., 2021 [89]	RCT	Iran	79 BD pt. recruited; 40 completed the study (24 in quetiapine group, and 20 in placebo group; mean age 36.45 ± 13.83 and 38.45 ± 14.60, respectively)	79	HDRS, YMRS, Y-BOCS	PR, TR	28/31
Saraf et al., 2017 [90]	Cross-sectional study	India	OCD (n = 171, mean age = 28.89 ± 9.52)	171	MINI, Y-BOCS, CGI, GAF, DSM-IV	PR, CI, PH	20/31
Saunders et al., 2012 [91]	Cross-sectional study	USA	BD, type I or Schizoaffective Disorder, Bipolar type (n = 736, mean age = 42 ± 12)	736	DIGS; DSM-IV	PR	19/31
Shabani et al., 2008 [92]	Case control study	Iran	OCD 78: BD-OCD (n = 39, mean age = 26.6 ± 7.23), OCD (n = 39, mean age = 30.1 ± 6.52)	78	SCID-CV; DSM-IV	PH	20/31
Shashidhara et al., 2015 [93]	Retrospective cohort study	India	BD (n = 396, mean age = 31.87 ± 11.1)	396	MINI, SCID-II, FIGS, Y-BOCS, YMRS, HAM-D, GAF, CGI-S; DSM-IV	PR, CI, HE, PH, TR	20/31
Simon et al., 2003 [94]	Case control study	USA	236 subjects: BD (n = 122, mean age = 40.8 ± 12.2)	122	SCID; DSM-IV	PR	23/31
Simon et al., 2004 [95]	Cross-sectional study	USA	BD (n = 475, mean age = 41.7 ± 12.8)	475	MINI; DSM-IV	PR, CI	23/31
Strakowski et al., 1992 [96]	Cross-sectional study	USA	BD (n = 41, mean age = 40.4 ± 11.7)	41	SCID; DSM-III-R	PR	22/31
Strakowski et al., 1998 [97]	Cross-sectional study	USA	BD, manic or mixed with psychosis (n = 77, mean age= 25 ± 6)	77	SCID-P; DSM-III-R	PR, CI	22/31
Tamam et al., 2002 [98]	Cross-sectional study	Turkey	BD-I in remission (n = 70, mean age = 33.4 ± 10.3)	70	SCID-CV; DSM-IV	PR	20/31
Tasdemir et al., 2015 [99]	Cross-sectional study	Turkey	BD (n = 70, mean age = 38.7 ± 11.1)	70	SCID, HAM-A, SCI-SAS, BDFQ, SASI, ASA; DSM-IV-TR	PR, CI	21/31
Timpano et al., 2012 [100]	Case control study	USA	OCD (n = 605, mean age = 39.2)	605	SCID-P; DSM-IV	PR, PH, CI	20/31
Tonna et al., 2021 [101]	Cross-sectional study	Italy	BD (n = 154), OCD (n = 11) (mean age 46 ± 11.74): euthymic (n = 62), hypomanic/manic (n = 34), depressive (n = 43), mixed (n = 26) state	165	Y-BOCS, HAM-D, YMRS, RRS; DSM-5	PR, PH, TR	22/31
Tukel et al., 2006 [102]	Case control study	Turkey	OCD (n = 115, age > 18)	117	SCID-CV for DSM-IV	PH, CI	21/31
Tukel et al., 2007 [103]	Case control study	Turkey	OCD (n = 128, mean age = 29.3 ± 10.8)	128	SCID-CV; DSM-IV	PR, CI	21/31
Zutshi et al., 2006 [104]	Case control study	India	BD in remission (n = 80, mean age = 30.06 ± 7.77)	80	SCID-CV; DSM-IV	PR	22/31
Zutshi et al., 2007 [105]	Case control study	India	OCD (n = 106, mean age: BD-OCD = 27.93 ± 6.71, OCD = 26.47 ± 7.38)	106	SCID-CV; DSM-IV	PH, HE, CI	20/31
Population-based studies: children, adolescents							
Alvarenga et al., 2015 [106]	Cross-sectional study	Brazil	2512 children (6–12 years old, mean age 8.86 ± 1.84): OCD (n = 77), OCS (n = 448)	77/448	FHS, DAWBA, SDQ, CBCL; DSM-IV	PR	23/31
Hofer et al., 2017 [107]	Prospective cohort study	Germany	3.021 subjects recruited age 14–24, assessed for up to 10 years: OCD (n = 210)	210	DIA-X/M-CIDI, DIA-X/M-CID OCD module; DSM-IV	PR	23/31
Hospital-based studies: children, adolescents							
Dilsaver et al., 2006 [108]	Case control study	USA	Latino adolescents (n = 313): BD (n = 115, mean age = 14.6 ± 1.5)	115	SCID-CV; DSM-IV	PR, CI	18/31
Joshi, Mick et al., 2010 [109]	Open label trial	USA	BD enrolled in the olanzapine trials (n = 52, mean age = 8.4 ± 3.1)	52	K-SADS-E; DSM-IV	TR	20/31
Joshi, Wozniak et al., 2010 [110]	Case control study	USA	OCD (n = 125, age range = 6–17), BD (n = 82, age range = 6–17)	207	K-SADS-E; DSM-III-R	PR, PH, CI	19/31
Masi et al., 2004 [111]	Case control study	Italy	BD, OCD, BD-OCD (n = 102, mean age = 14.2 ± 3.2)	102	DICA-R; DSM-IV	PH, CI	21/31
Masi et al., 2005 [112]	Prospective cohort study	Italy	OCD (n = 94, mean age = 13.6 ± 2.8)	94	DICA-R; DSM-IV	PR	20/31
Masi et al., 2007 [113]	Prospective cohort study	Italy	OCD (n = 120, mean age = 13.7 ± 2.8)	120	K-SADS-PL or DICA-R; DSM-IV	PR, PH, TR, CI	21/31
Masi et al., 2009 *** [114]	Case control study	Italy	OCD (n = 257, mean age = 13.6 ± 2.7)	257	K-SADS-PL; DSM-IV	PR, TR	22/31
Masi et al., 2010 *** [115]	Cross-sectional study	Italy	OCD (n = 257, mean age = 13.6 ± 2.7)	257	K-SADS-PL; DSM-IV	PR, PH	22/31
Masi et al., 2018 [116]	Prospective cohort study	Italy	BD (n = 172, mean age = 13.7 ± 2.9), OCD (n = 169, mean age = 13.2 ± 2.7), BD-OCD (n = 88, mean age = 14.2 ± 2.6)	429	K-SADS-PL, CGI, C-GAS; DSM-IV, DSM-IV-TR, DSM-5	PR, CI, PH, TR	23/31
Paul et al., 2015 [117]	Cross-sectional study	India	Patients aged <18 (n = 100): BD (n = 85, mean age 15.57 ± 1.75)	85	K-SADSPL, CY-BOCS, YMRS, CDI, HAM-A, BPRS-C; DSM-IV-TR	PR, CI, PH	22/31
Reddy et al., 2000 [118]	Cross-sectional study	India	OCD (n = 54, age = 16 or less)	54	DICA-R; DSM-III-R	PR	15/31
Tillman et al., 2003 [119]	Case control study	USA	BD (n = 93, mean age = 10.9 ± 2.6)	93	WASH-U-K-SADS; DSM-IV	PR	20/31

BD: bipolar disorder; BD-I: bipolar disorder type I; BD-II: bipolar disorder type II; BM: biological markers; CI: course of illness; CIDI: the WHO Composite International Diagnostic Interview; CYC: cyclothymic; DIA-X/M-CIDI: Munich Composite International Diagnostic Interview; DICA-R: Diagnostic Interview for Children and Adolescents—Revised; DIGS: Diagnostic Instrument for Genetic Studies; DIS: Diagnostic Interview Schedule; DSM: Diagnostic and Statistical Manual of Mental Disorders; HE: heredity; K-SADS-E: Kiddie Schedule for Affective Disorders and Schizophrenia for School-Age Children—Epidemiologic Version; K-SADS-PL: Schedule for Affective Disorders and Schizophrenia for School Age Children—Present and Lifetime Version; MDE: major depressive episode; MINI: Mini International Neuropsychiatric Interview; NS: not specified; OCD: obsessive–compulsive disorder; PH: phenomenology; PR: prevalence; Pt.: patients; SADS-L: Schedule for Affective Disorders and Schizophrenia, Lifetime Version; SCID: Structured Clinical Interview; SCID-CV: Structured Clinical Interview Clinical Version; SCID-P: Structured Clinical Interview, Patient Version; TR: treatment; WASH-U-K-SADS: Washington University in St. Louis Kiddie Schedule for Affective Disorders and Schizophrenia. * Checklist for measuring study quality developed by Downs and Black; ** They refer to the same study but were included separately, as one focused on prevalence of BD-OCD comorbidity and the other focused more in detail on BD-OCD family history, associations with substance abuse, and consequences of comorbidity; *** They refer to the same pt. sample.

**Table 2:** Lifetime prevalence of obsessive–compulsive disorder (OCD) comorbidity in bipolar disorder (BD) subjects.

BD Type	BD		BD-I	BD-II
References	N	%	%	%
Population-based studies				
Cederlöf et al., 2014 [24]	76	0.26		
Chen et al., 1995 [25]	167	21.0		
Faravelli et al., 2004 [26]	19	11.1		
Merikangas et al., 2007 [30]	408	13.6	25.2	20.8
Teh et al., 2020 [32]	105	27.8		
Hospital-based studies: adults				
Baptista et al., 2020 [34]	40	20	15.3	28.5
Bener et al., 2016 [36]	396	23.2		
Berkol et al., 2021 [37]	255	21.25		
Boylan et al., 2004 [39]	138	8.7		
Braverman et al., 2021a [41]	73	26		
Braverman et al., 2021b [42]	70	38.6		
Cassano et al., 1999 [44]	48			
Cosoff et al., 1998 [46]	20	30		
Craig et al., 2002 [47]	138	3.8		
Das et al., 2013 [48]	84	7.1	100	
de Filippis et al., 2018 [49]	48	54.2		
Di Salvo et al., 2020 [50]	201	20.3	38.3	59.7
Dilsaver et al., 2008 [52]	69		62.3	
Edmonds et al., 1998 [55]	55	1.8		
Henry et al., 2003 [58]	318	3		
Jeon et al., 2017 [62]	314	15.9		
Kazhungil et al., 2017 [63]	90	35	100	
Kemp et al., 2014 [65]	264	10.2		
Khan et al., 2019 [64]	469	7.5	100	
Khorshidian et al., 2023 [66]	64	100		
Kim et al., 2014 [67]	174	13.8	100	
Koyuncu et al., 2010 [68]	214	16.3	11.9	23.1
Kruger et al., 1995 [69]	37	35.1		
Kruger et al., 2000 [70]	143	7		100
Magalhaes et al., 2010 [73]	259	12.4		
Masi et al., 2018 [116]	52	33.8		
McElroy et al., 2001 [78]	288	9	9	10
Ozdemiroglu et al., 2017 [80]	80	40		
Pini et al., 1997 [85]	24			
Pini et al., 1999 [86]	125			
Sahraian et al., 2017 [87]	58	100	100	
Sahraian et al., 2018 [88]	56	100	100	
Sahraian et al., 2021 [89]	79	100	100	
Saunders et al., 2012 [91]	736	6		
Shashidhara et al., 2015 [93]	396	7.6	100	
Simon et al., 2003 [94]	122	13.4		
Simon et al., 2004 [95]	475	9.9	10.9	7.0
Strakowski et al., 1992 [96]	41	7.3		
Strakowski et al., 1998 [97]	77	16		
Tamam et al., 2002 [98]	70			
Tasdemir et al., 2015 [99]	70	13.2		
Tonna et al., 2021 [101]	165	93.3	81.2	18.8
Zutshi et al., 2006 [104]	80			
Hospital-based studies: children, adolescents				
Dilsaver et al., 2006 [108]	115	46.9		
Joshi, Wozniak et al., 2010 [109]	82	20.7		
Paul et al., 2015 [117]	85	2.4		
Tillman et al., 2003 [119]	93	24.7		

BD: bipolar disorder; BD-I: bipolar disorder type I; BD-II: bipolar disorder type II; N: total sample. Polarity of mood episodes is not specified in most studies.

**Table 3:** Lifetime prevalence of bipolar disorder (BD) comorbidity in obsessive–compulsive (OCD) subjects by BD type.

BD Type		BD	BD-I	BD-II
References	N	%	%	%
Population-based studies				
Abramovitch et al., 2019 [21]	316	3.2		
Adam et al., 2012 [22]	30	10.0		
Angst et al., 2004 [15]	30	53.3		30.0
Cederlöf et al., 2014 [24]	787	4.16		
Fineberg et al., 2013 [27]	30	40		
Fireman et al., 2001 [28]	1078 (adults) 198 (children and adolescents)	6.0 5.0		
Huang et al., 2014 [29]	1763 277	3.2 0.3		
Osland et al., 2018 [122]	267	18.2		
Subramaniam et al., 2020 [31]	217	8.8		
Hospital-based studies: adults				
Baptista et al., 2020 [34]	42	35.7	19	16.7
Benatti et al., 2014 [35]	75	9.3		
Bogetto et al., 1999 [38]	160			6.8
Bramante et al., 2021 [40]	601	17.1	6	11.1
de Filippis et al., 2018 [49]	46	43.5		
Diniz et al., 2004 [53]	161	9.0		
Domingues-Castro et al., 2019 [54]	74	7.8	42	53
Hantouche et al., 2003 [17]	628	11.0	3.0	8.0
Jakubovski et al., 2013 [61]	196	4.6		
Lensi et al., 1996 [72]	263		1.5	12.1
Marazziti et al., 2002 [76]	117	20.5	5.0	15.3
Masi et al., 2018 [116]	36	34.2		
Ozdemiroglu et al., 2017 [80]	93	34.4		
Perugi et al., 1997 [81]	345	15.7	2.0	13.6
Perugi et al., 1998 [82]	135	19.2	1.4	17.7
Perugi et al., 1999 [83]	79	21.5	3.8	17.7
Perugi et al., 2002 [84]	68	55.8	17.6	38.2
Saraf et al., 2017 [90]	171	4		
Timpano et al., 2012 [100]	605	13.1	8.4	4.6
Tukel et al., 2007 [103]	128	7.0		
Population-based studies: children, adolescents				
Alvarenga et al., 2015 [106]	77	1.3		
Hofer et al., 2017 [107]	210	4.5		
Hospital-based studies: children, adolescents				
Joshi, Wozniak et al., 2010 [109]	125	15.2		
Masi et al., 2005 [112]	94	24.4		
Masi et al., 2007 [113]	120	35.8	11.7	16.7
Masi et al., 2009 [114]	257	34.2		
Masi et al., 2010 [115]	257	34.2		
Reddy et al., 2000 [118]	54	1.8		

BD: bipolar disorder; BD-I: bipolar disorder type I; BD-II: bipolar disorder type II; N: total sample.

**Table 4:** Diagnostic validators: phenomenology, heredity, biological markers, and treatment.

References	Results
Phenomenology	
Bener et al., 2016 [36]	Lower mean HAM-D score in BD-OCD pt. compared to BD pt. (3.18 ± 0.59 vs. 3.74 ± 0.51, *p* = 0.016). No statistically significant difference in terms of YMRS score between BD-OCD pt. and BD pt.
Bramante et al., 2021 [40]	OCD pt. with forbidden thoughts symptoms presented higher rates of comorbid BD-I compared to OCD pt. without forbidden thoughts symptoms (7.6% vs. 2.9%, *p* = 0.023).
Braveman et al., 2021b [42]	Pt. with bipolar depression and comorbid OCD presented a significantly higher rate of body dysmorphic, hoarding, excoriation, and tic disorders compared to pt. with bipolar depression. No differences were found in the rate of trichotillomania.
de Filippis et al., 2018 [49]	No statistically significant difference in cognitive function among BD-OCD comorbidity and single diagnosis, including decision-making and cognitive flexibility, was found.
Dell’Osso et al., 2000 [51]	Patients with depression exhibited a greater prevalence of OCD compared to those experiencing mania.
Domingues-Castro et al., 2019 [54]	Compared to OCD pt., OCD-BD pt. more frequently presented “poor insight” (OR 1.98, 95% CI = 1.08–3.63), sensory phenomena (OR 1.90, 95% CI = 1.05–3.45), and higher severity of anxiety and depressive symptoms.
Hasler et al., 2005 [57]	Obsessions related to symmetry and compulsions involving repeating, counting, and ordering/arranging were linked to bipolar disorder (OR = 1.5, 95%; CI = 1.1–2.2).
Issler et al., 2005 [59]	A greater occurrence of aggression, symmetry, contamination, hoarding, and miscellaneous obsessions, as well as cleaning, checking, ordering, and various other compulsions, was observed in patients with comorbid BD and OCD compared to those without the comorbidity.
Jeon et al., 2017 [62]	In 65.4% of BD-OCD pt., obsessive–compulsive symptoms worsened or were confined to depressive episodes. Moreover, contamination obsession and checking compulsion were the most common types of obsessive–compulsive symptoms.
Joshi, Wozniak et al., 2010 [109]	Elevated frequencies of hoarding/saving obsessions (58% vs. 23%) and compulsions (63% vs. 20%) were observed in patients with comorbid BD and OCD compared to those without BD-OCD.
Kazhungil et al., 2017 [63]	A total of 25% of BD-OCD pt. predominantly had obsessions, 37.5% predominantly had compulsions, and 37.5% had mixed obsessions and compulsions. No significant differences between groups in HDRS and YMRS were found.
Khan et al., 2019 [64]	A majority of the BD-OCD pt. had OCD symptoms during a manic phase (40%) or in remission (37.1%), with contamination as the main theme (42.8%).
LaSalle-Ricci et al., 2006 [71]	A diagnosis of BD-I exhibited a significant and positive correlation with hoarding tendencies.
Mahasuar et al., 2011 [74]	Patients with comorbid OCD-BD demonstrated fewer pathological doubts (50% vs. 75%), an increased prevalence of pathological slowness (32% vs. 9%) and reassurance seeking (33% vs. 11%), and poorer insight into obsessive–compulsive symptoms (Y-BOCS-11: 1.11 ± 1.00 vs. 0.52 ± 0.71) compared to those without OCD-BD.
Maina et al., 2007 [75]	Elevated frequencies of hoarding (33.3% vs. 10.9%), sexual obsessions (42.9% vs. 19.7%), and repeating compulsions (71.4% vs. 42.6%) were observed in patients with comorbid OCD-BD compared to those without OCD-BD.
Marazziti et al., 2002 [76]	Patients affected by comorbid OCD-BD who had a positive history of repeated manic or hypomanic episodes exhibited a lower level of insight compared to patients with OCD alone.
Masi et al., 2004 [111]	Elevated frequencies of ordering compulsions (30% vs. 5.7%) were observed in patients with BD-OCD compared to those without BD-OCD.
Masi et al., 2007 [113]	Increased frequencies of hoarding obsessions and compulsions were observed in patients with OCD-BD compared to those without OCD-BD (14% vs. 2.6%).
Masi et al., 2010 [115]	An increased prevalence of BD was noted in patients with OCD who exhibited hoarding compulsions.
Masi et al., 2018 [116]	BD-OCD pt. presented a higher occurrence of BD-II compared to BD pt. Hoarding symptoms were more represented in BD-OCD pt. compared to OCD pt.
McElroy et al., 1995 [77]	Patients with episodes of mixed mania were found to be more prone to having comorbid OCD compared to those experiencing pure euphoric mania (21% vs. 4%).
Ozdemiroglu et al., 2017 [80]	Bipolarity did not have a specific effect on the phenomenology of OC symptoms.
Perugi et al., 1997 [81]	Elevated frequencies of sexual (41.1% vs. 20.3%) and religious obsessions (23.5% vs. 9.8%) were observed, along with a lower rate of checking rituals (56.8% vs. 73.4%), in patients with BD-OCD compared to those without BD-OCD.
Perugi et al., 2002 [84]	Patients with comorbid BD and OCD exhibited elevated frequencies of sexual obsessions (55.3% vs. 26.7%) and a decreased rate of ordering rituals (18.4% vs. 50.0%) compared to those without BD-OCD.
Shabani et al., 2008 [92]	Patients with BD-OCD demonstrated elevated frequencies of sexual obsessions (87.2% vs. 0.0%), religious obsessions (38.5% vs. 15.4%), and aggressive obsessions (35.9% vs. 7.7%), along with a reduced rate of contamination obsessions (64.1% vs. 84.6%), compared to those without BD-OCD. Additionally, BD-OCD patients exhibited a higher prevalence of miscellaneous compulsions (53.8% vs. 25.6%, *p* = 0.01) and a lower prevalence of washing compulsions (48.7% vs. 84.6%) compared to non-BD-OCD patients.
Shashidhara et al., 2015 [93]	BD-OCD pt. had significantly lower incidence of psychotic symptoms compared to BD pt. (53.6% vs. 30%, *p* = 0.01).
Timpano et al., 2012 [100]	Patients with OCD-BD exhibited more severe obsessive–compulsive symptoms, as indicated by a higher Y-BOCS total score (22.7 ± 9.8 vs. 18.2 ± 8.5), compared to those without OCD-BD.
Tonna et al., 2021 [101]	The severity of OCS was associated with the severity of depressive symptoms. The highest level of severity of OCS was observed in the mixed group, and the lowest scores were observed in the hypomanic/manic group.
Tukel et al., 2006 [102]	Patients with comorbid BD and OCD demonstrated elevated frequencies of symmetry/exactness obsessions (73.1% vs. 40.8%) and ordering/arranging compulsions (61.5% vs. 36.7%), along with lower rates of somatic obsessions (7.7% vs. 14.3%), compared to those without BD-OCD.
Zutshi et al., 2007 [105]	Patients suffering from BD-OCD showed less severe obsessive–compulsive symptoms, as indicated by a lower Y-BOCS total score (14.43 ± 7.27 vs. 25.53 ± 7.54), and lower rates of washing (32% vs. 62%), repeating (7% vs. 54%), and ordering (3% vs. 27%) compulsions compared to those without BD-OCD.
Heredity	
Angst et al., 2005 [16]	There were no statistically significant differences in family history regarding OCD, depression, and mania between individuals with obsessive–compulsive symptoms with or without comorbid BD.
Bener et al., 2016 [36]	A significantly higher proportion of BD pt. with (7.6%) and without OCD (17.8%) had a family history of BD (*p* = 0.030).
Cederlöf et al., 2014 [24]	OCD-unaffected first-, second-, and third-degree relatives of probands with OCD had a significantly increased risk for BD; the magnitude of this risk decreased as the genetic distance increased.
Domingues-Castro et al., 2019 [54]	OCD-BD pt. presenting comorbidity had more family history of affective symptoms compared to OCD pt. (depressive symptoms, OR 2.03, 95% CI = 1.13–3.65; agitation/euphoria symptoms OR 3.94, 95% CI = 2.37–6.54).
Kazhungil et al., 2017 [63]	In BD-OCD pt., first- and second-degree relatives had higher rates of BD-OCD and OCD but not of BD.
Khan et al., 2019 [64]	BD-I-OCD pt. had a higher incidence of OCD (2.9% vs. 0.2%, *p* = 0.008), as well as BD, in the family (20% vs. 9.2%).
Koyuncu et al., 2010 [68]	The frequency of OCD was higher in first-degree relatives of patients with comorbid BD and OCD compared to those without BD-OCD (45.7% vs. 5.7%). There were no statistically significant differences in the family history of BD.
Mahasuar et al., 2011 [74]	There were statistically non-significant trends indicating a higher prevalence of family history for mood disorders in patients with comorbid OCD and BD and a lower prevalence of family history for OCD compared to those without OCD-BD.
Ozdemiroglu et al., 2017 [80]	No difference in family history of OCD and BD between BD-OCD and BD pt.
Perugi et al., 2002 [84]	There were statistically non-significant trends suggesting a higher prevalence of family history for mood disorders in patients with BD-OCD and a lower prevalence of family history for OCD compared to those without BD-OCD.
Perugi et al., 1998 [83]	There was a positive correlation between episodic OCD and a family history of mood disorders compared to patients with continuous OCD (54.1% vs. 34.7%).
Shashidhara et al., 2015 [93]	Higher rates of OCD in first-degree relatives of BD-OCD pt. compared to BD pt. (6.7% vs. 0.3%, *p* = 0.02) were observed.
Zutshi et al., 2007 [105]	There was a higher prevalence of family history for mood disorders in BD-OCD compared to patients without BD-OCD (36% vs. 6%). Additionally, there was a lower prevalence of family history for OCD in BD-OCD patients compared to non-BD-OCD patients (0.0% vs. 21%).
Biological markers	
Cannon et al., 2006 [43]	There was a higher binding potential of 5-HTT in OCD-BD compared to patients without OCD-BD. This was observed in the insula, posterior cingulate cortex, subgenual anterior cingulate cortex, and dorsal cingulate cortex.
Treatment	
Benatti et al., 2014 [35]	In OCD-BD pt., 86% were on atypical antipsychotic treatment (alone: 71%; in association with lithium/valproate: 15%) and 14% were on antidepressant treatment in association with lithium or valproate.
Domingues-Castro et al., 2019 [54]	OCD-BD pt. were more commonly treated with psychotherapy compared to OCD pt. (OR 1.80, 95% CI = 1.03–3.12).
Jeon et al., 2017 [62]	A drug-induced (hypo)manic switch was observed in more than 60% of the BD-OCD pt. who were previously exposed to antidepressants. None of the BD-OCD pt. were taking antidepressants for OCD in the specialty clinics at that time.
Joshi, Mick et al., 2010 [109]	OCD-BD exhibited a lower treatment response compared to patients without OCD-BD, as evidenced by a smaller mean reduction in YMRS scores (−5.9 ± 13.1 vs. −13.7 ± 18.8), a lower percentage of ≥30% reduction (25% vs. 63%), and a lower proportion achieving a CGI-S Improvement score ≤ 2 (25% vs. 68%). However, no statistically significant differences were reported in the rates of dropouts or adverse effects.
Kazhungil et al., 2017 [63]	The number of failed trials of mood stabilizers, antipsychotics, and antidepressants used in their lifetime did not differ between BD-OCD pt. and BD pt.
Khan et al., 2019 [64]	A total of 60% of BD-OCD pt. were on an antidepressant/anxiolytic, with a mood stabilizer or antipsychotic or both, as compared to 45.8% of BD-I pt. who received antidepressants/anxiolytics. A higher number of BD-OCD pt. received SSRIs/antidepressants/anxiolytics.
Khorshidian et al., 2023 [66]	Both aripiprazole and risperidone were effective as an adjunctive therapy, with valproate sodium used for treating OCD in patients with BD without any serious and life-threatening adverse effect. Aripiprazole was more effective than Risperidone in treating OCD in BD (*p* < 0.001).
Kim et al., 2014 [67]	BD-OCD pt. were significantly associated with lower remission rates if treated with olanzapine ± other mood stabilizer compared to a mood stabilizer alone.
Masi et al., 2007 [113]	All patients with OCD received treatment with selective serotonin reuptake inhibitors (SRIs) such as sertraline, fluvoxamine, fluoxetine, paroxetine, citalopram, or clomipramine, either in mono- or polytherapy (89.2%), along with mood stabilizers (valproic acid, lithium) in 37.5% and antipsychotics (risperidone, olanzapine, quetiapine) in 30.8%. Patients with OCD-BD were more likely to receive mood stabilizers compared to those without OCD-BD (86.0% vs. 10.4%). Additionally, 69.8% of OCD-BD patients were treated with SRIs. There was a higher rate of non-responders to pharmacological treatment in OCD-BD patients compared to those without OCD-BD (54.7% vs. 25.6%).
Masi et al., 2009 [114]	Patients suffering from OCD-BD were more likely to receive polypharmacy compared to those taking selective serotonin reuptake inhibitors (SRIs) alone (51.1% vs. 5.6%).
Masi et al., 2018 [116]	BD-OCD pt. more frequently received psychotherapy and second-generation antipsychotics compared to BD and OCD pt., and they presented the poorest outcome in terms of response to treatments.
Ozdemiroglu et al., 2017 [80]	All the pt. recruited were under mood stabilizers, antidepressants, or antipsychotics.
Perugi et al., 2002 [84]	In patients affected by BD-OCD, clomipramine and, to a lesser extent, SSRIs were associated with a higher rate of manic/hypomanic switches compared to those without BD-OCD (clomipramine: 39.1% vs. 4.1%; SSRIs: 13.9% vs. 0.0%). There was a more frequent occurrence of pharmacologic mania/hypomania in BD-OCD patients who did not concurrently receive mood stabilizers (38.7% vs. 8.8%). Polypharmacologic treatments were required in 31.6% (a combination of mood stabilizers, lithium, plus antiepilectics) and 10.5% (a combination of mood stabilizers with atypical antipsychotics, clozapine, olanzapine, and risperidone) of the BD-OCD patients.
Sahraian et al., 2017 [87]	Memantine, as an adjunctive agent, was more effective than placebo in decreasing the OC symptoms (with more than 34% decrease in the mean Y-BOCS score) in BD-I-OCD pt. in the manic phase.
Sahraian et al., 2018 [88]	Aripiprazole showed effectiveness in reducing OC symptoms (with more than 34% decline in the Y-BOCS score) in BD-OCD pt. compared to placebo (91.30% vs. 4.34%).
Sahraian et al., 2021 [89]	Quetiapine showed greater effectiveness than placebo in reducing OC symptoms (with more than 34% decline in the Y-BOCS score (*p* < 0.001)) in BD-OCD pt. (Y-BOCS score from 24.37 to 15.26 (*p* < 0.001)) compared to placebo (Y-BOCS from 24.21 to 23.94 (*p* = 1.97)). No serious adverse effects were reported in the two groups.
Saraf et al., 2016 [90]	OCD-BD pt. received more SSRI trials at baseline; 86% of OCD-BD pt. were treated with adjunctive mood stabiliser, 67% were treated with lithium, and 33% were treated with valproate.
Shashidhara et al., 2015 [93]	No significant difference in the pattern of use of various medications and their combinations was observed between BD-OCD pt. and BD pt. None of the OCD pt. received antidepressants during the index mood episodes for which they were hospitalized.
Tonna et al., 2021 [101]	All pt. received one or more mood stabilizers. Antipsychotics were added in 94.1% of hypomanic/manic pt., in 84.6% of mixed pt., in 23.2% of depressed pt. and in 51.6% of euthymic pt. Antidepressants were added in 88.3% of depressed pt., in 15.3% of mixed pt. and in 16.1% of euthymic pt.

BD: bipolar disorder; BD-I: bipolar disorder type I; BD-II: bipolar disorder type II; HSCT: Hayling Sentence Completion Test; IGT: Iowa Gambling Task; OCD: obsessive–compulsive disorder; OCS: obsessive–compulsive syndrome; pt.: patients; vs.: versus; Y-BOCS: Yale–Brown Obsessive–Compulsive Scale; YMRS: Young Mania Rating Scale; CGI-S: Clinical Global Impression Severity; RCFT: Rey–Osterrieth Complex Figure Test; SSRIs: Serotonin Reuptake Inhibitors; TMT: Trial Making Task; WCST: Wisconsin Card Sorting Test; 5-HTT: Serotonin Transport Protein. Differences statistically significant (*p* < 0.05). The colors refer to the primary population diagnosis whether BD (green), OCD (blue), or not known (yellow).

**Table 5:** Diagnostic validator: course of illness.

References	Results
Age and type of onset	
Anholt et al., 2014 [33]	Early age of OCD onset pt. presented higher rates of lifetime BD co-morbidity.
Cederlöf et al., 2014 [24]	The risk of receiving a diagnosis of BD after an initial diagnosis of OCD was much greater (around 12 times, with a median of 2.7 years between diagnoses (IR = 3.5)) than the risk of receiving a diagnosis of OCD after an initial diagnosis of BD (around 1.2 times, with a median time between diagnoses of 1.1 years (IR = 2.0)).
Chen et al., 1995 [25]	The average onset age of OCD and BD was similar.
Jeon et al., 2017 [62]	BD-OCD pt. presented earlier age at onset compared to BD pt. In 46.2% of comorbid pt., OCD onset preceded BD, whereas in 34.6%, BD preceded OCD, and in 19.2%, OCD began during the first mood episode.
Kazhungil et al., 2017 [63]	No significant difference in age was observed at the onset of BD between BD-OCD pt. and BD pt.
Maina et al., 2007 [75]	In the majority of cases (52.4%), the onset of OCD in patients with comorbid BD occurred simultaneously with the first mood episode rather than preceding it (38.1%) or following it (9.5%).
Masi et al., 2018 [116]	Age at onset of BD and OCD were not different between BD and OCD pt. and BD-OCD pt.
Ortiz et al., 2011 [79]	Comorbid OCD was associated with an early onset of BD and an early onset of depressive episodes in patients with BD.
Ozdemiroglu et al., 2017 [80]	Age at onset of BD was found to be earlier in BD-OCD pt. compared to BD pt. (23.9 vs. 29.2); age of onset of BD, compared to the age of onset of OCD, was found to be earlier in BD-OCD pt. (23.9 vs. 30). The first affective episode was major depression in half of BD-OCD pt.
Paul et al., 2015 [117]	OCD symptoms pre-dated the onset of mood disorder in all BD-OCD pt. compared to BD pt.
Perugi et al., 1997 [81]	OCD-BD showed a greater tendency toward a gradual onset compared to those without OCD-BD (68.5% vs. 49.0%).
Perugi et al., 2002 [84]	In the majority of cases (52.6%), the onset of OCD in patients with BD-OCD occurred simultaneously with the first mood episode, rather than preceding it (31.6%) or following it (15.8%).
Saraf et al., 2016 [90]	No difference between OCD-BD and OCD pt. with respect to age of onset of OCD was found.
Shashidhara et al., 2015 [93]	No significant difference was found in age at the onset of BD between BD-OCD pt. and BD pt.
Zutshi et al., 2007 [105]	In the majority of cases (54%), patients with OCD-BD experienced the onset of OCD before the onset of BD.
Course of illness	
Bener et al., 2016 [36]	BD pt. presented a longer duration of illness compared to BD-OCD pt. (10.96 ± 3.81 vs. 9.06 ± 3.32).
de Filippis et al., 2018 [49]	No differences in illness duration, number of depressive and manic episodes among BD, OCD and BD-OCD pt. were found.
Fineberg et al., 2013 [27]	OCD-BD exhibited a longer total illness duration (the time gap from the first manifestation until the last manifestation) compared to patients with only OCD (OR = 2.53; 95%; CI: 0.92–6.96).
Goes et al., 2012 [56]	Patients suffering from BD-OCD experienced a greater number of depressive episodes compared to those without BD-OCD (19.7 vs. 10.3).
Hofer et al., 2017 [107]	Prior OCD was associated with an increased risk of BD (OR = 6.9; 95% CI: 2.8–17.3).
Issler et al., 2005 [59]	A greater percentage of BD-OCD patients experienced a chronic course of OCD compared to those without BD-OCD (86.7% vs. 13.3%). ^(b)^
Issler et al., 2010 [60]	BD-OCD had a greater number of depressive episodes compared to those without BD-OCD (8.9 ± 4.2 vs. 4.1 ± 2.7). Additionally, there was a higher rate of a chronic course of bipolar disorder in patients with BD-OCD compared to those without BD-OCD (66.7% vs. 20%).
Jeon et al., 2017 [62]	No statistically significant differences between the BD-OCD pt. and BD pt. in terms of duration of illness, number of previous episodes, type of the first epsiode, and the most recent episode were found.
Kazhungil et al., 2017 [63]	BD-OCD pt. presented higher rates of manic and depressive episodes compared to BD pt.
Khan et al., 2019 [64]	BD-I-OCD pt. presented a longer duration of illness compared to BD pt.
Kim et al., 2014 [67]	Comorbid OCD was associated with lower remission rates in BD-I pt. both in the first and in the second year of the study evaluation compared to BD-I pt.
Koyuncu et al., 2010 [68]	BD-OCD exhibited a higher rate of a chronic course of BD compared to those without BD-OCD (17.1% vs. 0.0%). ^(c)^
Mahasuar et al., 2011 [74]	An episodic course was more prevalent in patients affected by OCD-BD compared to those without comorbidity (53% vs. 12%). On the other hand, higher rates of a continuous course (40% vs. 35%) or a subclinical course (32% vs. 6%) were observed in patients without OCD-BD compared to those with OCD-BD. ^(a),(b)^
Ozdemiroglu et al., 2017 [80]	A more episodic course of OCD (34.4% vs. 14.8%) was higher in BD-OCD pt. compared to OCD pt.; rates of rapid cycling (21.9% vs. 6.3%) and seasonality (50% vs. 20.8%) were found to be higher in BD-OCD pt. compared to BD pt.
Perugi et al., 1997 [81]	OCD-BD showed a greater inclination toward an episodic course of OCD symptoms compared to those without OCD-BD (42.6% vs. 26.9%). ^(a)^
Perugi et al., 1998 [82]	There were statistically non-significant trends indicating a higher rate of an episodic course of OCD in patients with comorbid BD-II compared to those without comorbidity. ^(a)^
Perugi et al., 2002 [84]	BD-OCD exhibited a higher rate of an episodic course of OCD compared to those without BD-OCD (52.6% vs. 16.7%). ^(a)^
Saraf et al., 2016 [90]	Episodic OCD was significantly overrepresented in OCD-BD pt. compared to OCD pt. (29% vs. 2%). All OCD-BD pt. presented a deterioration of OCD during depression; 86% reported improvement during mania/hypomania, whereas 14% reported complete remission during this phase.
Shashidhara et al., 2015 [93]	No significant difference in the total illness duration between BD-OCD pt. and BD pt. was found.
Strakowski et al., 1998 [96]	In 44% of patients with BD-OCD observed over a 12-month period following their initial hospitalization, BD and OCD exhibited cyclical patterns concurrently.
Tukel et al., 2006 [102]	A greater percentage of OCD-BD pt. experienced an episodic course of OCD compared to those without OCD-BD (42.3% vs. 10.9%). ^(a)^
Tukel et al., 2007 [103]	Patients with episodic OCD exhibited a higher frequency of BD compared to those with chronic OCD (20.8% vs. 3.8%). ^(a)^
Zutshi et al., 2007 [105]	There was a higher rate of an episodic course of OCD in patients with OCD-BD compared to patients without the comorbidity (75% vs. 3%). Conversely, there was a higher rate of a chronic course of OCD in patients without OCD-BD compared to those with comorbidity (97% vs. 14%). Among OCD-BD patients, the majority (78%) experienced OCD either exclusively during depressive episodes or reported worsening of OCD symptoms during depression. Improvement in obsessive–compulsive symptoms was noted in 64% of patients during manic/hypomanic episodes. ^(a),(b)^
Global functioning and quality of life	
Angst et al., 2005 [16]	There was a statistically significant difference in global impairment (100.0% vs. 87.5%) and work impairment (100.0% vs. 78.1%) between OCD-BD pt. and those with “pure” OCD.
Bener et al., 2016 [36]	Higher CGI (4.69 ± 0.66 vs. 4.12 ± 0.76) and GAF (39.67 ± 5.32 vs. 37.11 ± 6.86) scores were found in BD pt. compared to BD-OCD pt.
Carta et al., 2020 [23]	OCD-BDS pt. presented significantly poorer QoL compared to OCD pt. (33.6 ± 6.7 vs. 36.1 ± 7.1).
Centorrino et al., 2006 [45]	There were no statistically significant differences in terms of GAF and CGI-S scores between patients with “pure” OCD and BD and those with comorbidity.
Fineberg et al., 2013 [27]	Relationship issues were more frequently reported in OCD-BD pt. compared to OCD pt. (separation/divorce, OR = 4.11; 95% CI: 1.13–14.89; problems with partner, OR = 5.04; 95% CI: 1.27–20.10).
Joshi, Wozniak et al., 2010 [109]	Patients with BD-OCD and those with OCD-BD exhibited lower levels of lifetime and current functioning on the GAF scale compared to patients without a comorbidity.
Kazhungil et al., 2017 [63]	BD-OCD pt. showed lower GAF scores and more dysfunctional impairment compared to BD pt.
Khan et al., 2017 [64]	BD-I-OCD pt. presented a lower level of education and higher divorce rates compared to BD-I pt.
Magalhaes et al., 2010 [73]	Patients with BD-OCD scored lower on all domains of the WHOQOL questionnaire, including physical, psychological, social, and environmental domains, compared to those without OCD-BD
Mahasuar et al., 2011 [74]	There were no statistically significant differences in GAF, CGI-S, and CGI-I scores between patients with OCD with or without comorbid BD.
Masi et al., 2004 [111]	There was a higher mean global severity score, measured during the first visit using the CGI-S, in patients with BD-OCD compared to those with only OCD (4.80 ± 0.7 vs. 4.4 ± 0.5). The scores were similar in patients with BD and those with BD-OCD (5.0 ± 0.8 and 4.80 ± 0.7, respectively). Furthermore, there were higher rates of severity at the end of the follow-up in patients with BD and BD-OCD compared to those with obsessive–compulsive disorder alone (3.1 ± 1.0 and 2.7 ± 1.0, respectively, vs. 2.0 ± 0.7).
Masi et al., 2007 [113]	OCD-BD exhibited greater functional impairment (C-GAS) at the baseline (43.1 ± 8.7 vs. 46.4 ± 9.0) and greater severity (CGI-S) both at the baseline and at the end of the follow-up compared to those without OCD-BD.
Shashidhara et al., 2015 [93]	BD-OCD pt. presented significantly lower GAF scores (41.9 vs. 35.8) and higher rates of unemployment (10% vs. 5%) compared to BD pt.
Simon et al., 2004 [94]	Having a current anxiety disorder (excluding OCD) in patients with BD was linked to lower functioning (measured by LIFE-RIFT) and a lower quality of life (Q-LES-Q).
Timpano et al., 2012 [100]	There were no statistically significant differences in the GAF total score between patients with OCD with or without comorbid BD.
Suicide ideations and attempts	
Chen et al., 1995 [25]	Patients with BD-OCD had higher lifetime rates of “thoughts of suicide,” “suicide attempts,” “thoughts of death,” and “wanting to die” compared to those without BD-OCD.
Di Salvo et al., 2020 [50]	No significant differences were found comparing lifetime suicide attempts rates between BD pts with or without comorbid OCD. In the BD-OCD group, more pt. performed suicide attempts with violent methods (48.3% vs. 28.7%), with a correlation between comorbid OCD and male gender and violent suicide attempts.
Dilsaver et al., 2006 [52]	BD-OCD was associated with a 2.4-fold increase in the odds of suicidal ideation compared to patients without BD-OCD (95% CI = 1.0–5.8).
Domingues-Castro et al., 2019 [54]	OCD-BD pt. presented a higher suicide risk compared to OCD pt., with more suicide ideations (OR 2.08, 95% CI = 1.26–3.43), plans (OR 2.80, 95% CI = 1.66–4.72), and attempts (OR 2.64, 95% CI = 1.41–4.91).
Fineberg et al., 2013 [27]	Suicidal acts were more commonly reported in patients with OCD-BD compared to those with only OCD (OR = 10.38; 95%; CI: 3.14–34.35).
Goes et al., 2012 [56]	BD-OCD had higher rates of a history of suicide attempts compared to those without BD-OCD (48.3% vs. 29.6%).
Jeon et al., 2017 [62]	No statistically significant differences were observed between BD-OCD pt. and BD pt. in terms of a history of suicide attempts.
Kazhungil et al., 2017 [63]	BD-OCD pt. presented higher rates of suicidal attempts compared to BD pt.
Koyuncu et al., 2010 [68]	There were no statistically significant differences between pt. with BD-OCD and pt. without BD-OCD in terms of lifetime suicide attempts.
Kruger et al., 2000 [70]	There was a higher incidence of prior suicide attempts in patients with BD-OCD compared to those without BD-OCD (90% vs. 38%).
Magalhaes et al., 2010 [73]	Patients with BD-OCD had higher rates of a history of suicide attempts compared to those without BD-OCD (70% vs. 35%).
Ozdemiroglu et al., 2017 [80]	A previous history of suicidal attempts was more likely to be higher in BD-OCD pt. (46.9%) compared to BD pt. (20.8%) and OCD pt. (11.5%).
Saraf et al., 2016 [90]	OCD-BD pt. presented a higher rate of lifetime suicide attempts compared to OCD pt. (57% vs. 14%).
Simon et al., 2004 [95]	Patients with BD-OCD experienced higher rates of a history of suicide attempts compared to those without the comorbidity.
Hospitalization	
Bener et al., 2016 [36]	No statistically significant difference was observed in the number of hospitalizations between BD-OCD pt. and BD pt.
Centorrino et al., 2006 [45]	Re-hospitalization rates were comparable between patients affected by BD-OCD and those with BD alone, but they were 2.9 times more frequent in BD-OCD patients compared to those without comorbidity.
Joshi, Wozniak et al., 2010 [109]	There were higher rates of hospitalization in BD-OCD and OCD-BD compared to patients without comorbidity (BD-OCD: 41.2% vs. 10.4%; OCD-BD: 31.6% vs. 10.4%).
Kazhungil et al., 2017 [63]	BD-OCD pt. presented higher rates of hospitalizations compared to BD pt.
Kim et al., 2014 [67]	BD-I-OCD pt. were more likely to be hospitalized during the 24-month study period compared to BD-I pt. (54.2% vs. 35.3%), (difference statistically significant).
Mahasuar et al., 2011 [74]	There was a higher total number of hospitalizations in patients with OCD-BD compared to those without OCD-BD (1.55 ± 1.70 vs. 0.16 ± 0.41).
Masi et al., 2007 [113]	There was a higher frequency of the need for hospitalization in patients with OCD-BD compared to those without OCD-BD (62.8% vs. 28.6%).
Shashidhara et al., 2015 [93]	No statistically significant difference was found in the number of hospitalizations between BD-OCD pt. and BD pt.
Timpano et al., 2012 [100]	There was a more frequent requirement for hospitalization in patients with the BD-OCD comorbidity compared to those without OCD-BD (58.2% vs. 13.8%).
Substance and alcohol abuse	
Angst et al., 2005 [16]	Patients with obsessive–compulsive symptoms and comorbid BD (OCS-BD) exhibited higher rates of substance abuse/dependence (50% vs. 31.3%) and alcohol abuse/dependence (37.5% vs. 18.8%) compared to those with “pure” obsessive–compulsive symptoms (OCS pt.).
Boylan et al., 2004 [39]	BD-OCD had higher rates of past substance abuse or dependence compared to pt. without BD-OCD (50% vs. 20%).
Chen et al., 1995 [25]	Pt. with OCD-BD comorbidity exhibited rates of alcohol and drug abuse of 31.4% and 37.1%, respectively. Non-comorbid patients had rates of alcohol and drug abuse of 36.4% and 28.2%, respectively.
Fineberg et al., 2013 [27]	The occurrence of substance abuse disorders was more commonly reported in patients with OCD-BD compared to those with only OCD (OR = 3.65; 95%; CI 1.34–9.94).
Magalhaes et al., 2010 [73]	BD-OCD pt. had higher rates of lifetime alcohol dependence compared to pt. without BD-OCD (31% vs. 10%).
Maina et al., 2007 [75]	There was a significant association between OCD-BD and substance use disorders (28.6% vs. 4.9%) compared to pt. without OCD-BD.
Perugi et al., 2002 [84]	There was a stronger association between BD-OCD and the use of sedatives, nicotine, alcohol, and coffee (*p* = 0.03) compared to patients without this comorbidity.
Timpano et al., 2012 [100]	There was a robust association between OCD-BD and substance use disorders (OR 3.18, 95%; CI = 1.81–5.58), as well as alcohol use disorders (odds ratio 2.21, 95% CI = 1.34–3.65), compared to pt. without OCD-BD.
Other psychiatric comorbidities	
Domingues-Castro et al., 2019 [54]	BD-OCD pt. presented higher panic disorder with agoraphobia (OR 2.54, 95% CI = 1.53–4.20) and impulse control disorders (OR 2.41, 95% CI = 1.49–3.92) compared to OCD pt.
Issler et al., 2010 [60]	There were higher prevalence rates of any anxiety disorder other than OCD (93.3% vs. 53.3%), impulse control disorders (60% vs. 13.3%), EDs (33.3% vs. 0%), and tic disorders (33.3% vs. 0%) in patients with BD-OCD compared to those without BD-OCD.
Jeon et al., 2017 [62]	BD-OCD pt. presented a higher rate of comorbid panic disorder and eating disorders compared to BD pt.
Maina et al., 2007 [75]	There were higher rates of at least one Cluster A personality disorder (*p* = 0.027), at least one Cluster B personality disorder, and narcissistic and antisocial personality disorders in patients with OCD-BD compared to those without OCD-BD.
Masi et al., 2004 [111]	There were higher rates of ADHD-CD (51.3% vs. 20.0%) and CD (29.7% vs. 13.3%) in patients with BD compared to those with BD-OCD. Conversely, there were lower rates of ADHD (2.8% vs. 16.7%) and higher rates of GAD (77.1% vs. 16.7%) in patients with OCD compared to those with comorbid BD-OCD.
Masi et al., 2007 [113]	Patients with OCD-BD had higher comorbidity rates with ADHD and ODD and lower comorbidity rates with GAD.
Perugi et al., 2002 [84]	There were higher rates of current comorbidity with panic disorder/agoraphobia in patients with BD-OCD compared to those without BD-OCD (52.6% vs. 16.7%).
Shashidhara et al., 2015 [93]	BD-OCD pt. presented higher rates of comorbid social anxiety (10% vs. 1.4%) and anxious avoidant personality disorder (26.7% vs. 2.7%).
Tasdemir et al., 2015 [99]	Although not statistically significant, BD-OCD comorbidity was more common in BD pt. with adult separation anxiety disorder.
Timpano et al., 2012 [100]	Patients with OCD-BD were more than twice as likely to also be diagnosed with panic disorder (OR 2.26, 95% CI = 1.38–3.71), agoraphobia (OR 2.30, 95% CI = 1.35–3.91), PTSD (OR 2.85, 95% CI = 1.49–5.45), and an ED (OR 2.03, *p* < 0.001, 95% CI = 1.14–3.60) compared to patients without the comorbidity.
Medical comorbidities	
Kemp et al., 2014 [65]	BD-OCD pt. presented a higher rate of medical comorbidity compared to BD pt. without OCD (14.4% vs. 5.6%).
Khan et al., 2019 [64]	BD-I-OCD pt. presented fewer medical comorbidities compared to BD-I pt. but a higher frequency of cardiovascular conditions.

BD: bipolar disorder; BD-I: bipolar disorder type I; BD-II: bipolar disorder type II; BDS, Bipolar disorder spectrum; OCD: obsessive–compulsive disorder; OCS: obsessive–compulsive syndrome; pt.: patients; vs.: versus; GAD: generalized anxiety disorder; PD: panic disorder; PTSD: post-traumatic stress disorder; ED: eating disorder; ADHD: attention deficit hyperactivity disorder; CD: conduct disorder; GAF: Global Assessment of Functioning; CGI-S: Clinical Global Impression Severity; CGI-I: Clinical Global Impression Improvement; C-GAS: Children’s Global Assessment Scale; WHOQOL: the World Health Organization Quality of Life Brief; Q-LES-Q: Quality of Life Enjoyment and Satisfaction Questionnaire; LIFE-RIFT: Range of Impaired Functioning Tool; ^(a)^ Episodic OCD: at least one circumscribed symptom-free interval (six months) was present; ^(b)^ Chronic OCD: if symptoms persisted for most of the course, causing significant distress and impairment in functioning; ^(c)^ Chronic BD: if all criteria for a major mood episode were met continuously for at least two years. Statistically significant differences (*p* < 0.05). The colors refer to the primary population diagnosis, whether BD (green), OCD (blue), or not known (yellow).

## Data Availability

No new data were created or analyzed in this study. Data sharing is not applicable to this article.

## Supplementary Materials

The following supporting information can be downloaded at: https://www.mdpi.com/article/10.3390/jcm13051230/s1, Table S1: PRISMA Checklist; Table S2: MEDLINE search strategy; Table S3: Reference list of papers included in the review.
