# Supplementary material for: Obsessive–Compulsive Disorder as an Epiphenomenon of Comorbid Bipolar Disorder? An Updated Systematic Review
Source: J Clin Med. 2024 Feb 21;13(5):1230. doi: 10.3390/jcm13051230 (PMC10931838; doi:10.3390/jcm13051230)
Supplement: Supplementary file 1 [file jcm-13-01230-s001.zip › jcm-2776269-supplementary.pdf]

Supplementary Table S1. PRISMA Checklist.

Section and Topic	Item #	Checklist item	Location where item is reported
**TITLE**			
Title	1	Identify the report as a systematic review.	1
**ABSTRACT**			
Abstract	2	See the PRISMA 2020 for Abstracts checklist.	1
**INTRODUCTION**			
Rationale	3	Describe the rationale for the review in the context of existing knowledge.	2
Objectives	4	Provide an explicit statement of the objective(s) or question(s) the review addresses.	2
**METHODS**			
Eligibility criteria	5	Specify the inclusion and exclusion criteria for the review and how studies were grouped for the syntheses.	2-3
Information sources	6	Specify all databases, registers, websites, organisations, reference lists and other sources searched or consulted to identify studies. Specify the date when each source was last searched or consulted.	2
Search strategy	7	Present the full search strategies for all databases, registers and websites, including any filters and limits used.	2
Selection process	8	Specify the methods used to decide whether a study met the inclusion criteria of the review, including how many reviewers screened each record and each report retrieved, whether they worked independently, and if applicable, details of automation tools used in the process.	2
Data collection process	9	Specify the methods used to collect data from reports, including how many reviewers collected data from each report, whether they worked independently, any processes for obtaining or confirming data from study investigators, and if applicable, details of automation tools used in the process.	2
Data items	10a	List and define all outcomes for which data were sought. Specify whether all results that were compatible with each outcome domain in each study were sought (e.g. for all measures, time points, analyses), and if not, the methods used to decide which results to collect.	3
	10b	List and define all other variables for which data were sought (e.g. participant and intervention characteristics, funding sources). Describe any assumptions made about any missing or unclear information.	3
Study risk of bias assessment	11	Specify the methods used to assess risk of bias in the included studies, including details of the tool(s) used, how many reviewers assessed each study and whether they worked independently, and if applicable, details of automation tools used in the process.	2-3
Effect measures	12	Specify for each outcome the effect measure(s) (e.g. risk ratio, mean difference) used in the synthesis or presentation of results.	NA
Synthesis	13a	Describe the processes used to decide which studies were eligible for each synthesis (e.g. tabulating the study intervention	2-3

Section and Topic	Item #	Checklist item	Location where item is reported
methods		characteristics and comparing against the planned groups for each synthesis (item #5)).	
	13b	Describe any methods required to prepare the data for presentation or synthesis, such as handling of missing summary statistics, or data conversions.	2-3
	13c	Describe any methods used to tabulate or visually display results of individual studies and syntheses.	4-12
	13d	Describe any methods used to synthesize results and provide a rationale for the choice(s). If meta-analysis was performed, describe the model(s), method(s) to identify the presence and extent of statistical heterogeneity, and software package(s) used.	4-12
	13e	Describe any methods used to explore possible causes of heterogeneity among study results (e.g. subgroup analysis, meta-regression).	NA
	13f	Describe any sensitivity analyses conducted to assess robustness of the synthesized results.	NA
Reporting bias assessment	14	Describe any methods used to assess risk of bias due to missing results in a synthesis (arising from reporting biases).	NA
Certainty assessment	15	Describe any methods used to assess certainty (or confidence) in the body of evidence for an outcome.	NA
**RESULTS**			
Study selection	16a	Describe the results of the search and selection process, from the number of records identified in the search to the number of studies included in the review, ideally using a flow diagram.	3-4
	16b	Cite studies that might appear to meet the inclusion criteria, but which were excluded, and explain why they were excluded.	3-4
Study characteristics	17	Cite each included study and present its characteristics.	4-12
Risk of bias in studies	18	Present assessments of risk of bias for each included study.	4-12
Results of individual studies	19	For all outcomes, present, for each study: (a) summary statistics for each group (where appropriate) and (b) an effect estimate and its precision (e.g. confidence/credible interval), ideally using structured tables or plots.	12-29
Results of syntheses	20a	For each synthesis, briefly summarise the characteristics and risk of bias among contributing studies.	12-29
	20b	Present results of all statistical syntheses conducted. If meta-analysis was done, present for each the summary estimate and its precision (e.g. confidence/credible interval) and measures of statistical heterogeneity. If comparing groups, describe the direction of the effect.	12-29
	20c	Present results of all investigations of possible causes of heterogeneity among study results.	12-29

Section and Topic	Item #	Checklist item	Location where item is reported
	20d	Present results of all sensitivity analyses conducted to assess the robustness of the synthesized results.	NA
Reporting biases	21	Present assessments of risk of bias due to missing results (arising from reporting biases) for each synthesis assessed.	NA
Certainty of evidence	22	Present assessments of certainty (or confidence) in the body of evidence for each outcome assessed.	NA
**DISCUSSION**			
Discussion	23a	Provide a general interpretation of the results in the context of other evidence.	29-31
	23b	Discuss any limitations of the evidence included in the review.	31-32
	23c	Discuss any limitations of the review processes used.	31-32
	23d	Discuss implications of the results for practice, policy, and future research.	29-31
**OTHER INFORMATION**			
Registration and protocol	24a	Provide registration information for the review, including register name and registration number, or state that the review was not registered.	2
	24b	Indicate where the review protocol can be accessed, or state that a protocol was not prepared.	2
	24c	Describe and explain any amendments to information provided at registration or in the protocol.	2
Support	25	Describe sources of financial or non-financial support for the review, and the role of the funders or sponsors in the review.	32
Competing interests	26	Declare any competing interests of review authors.	33
Availability of data, code and other materials	27	Report which of the following are publicly available and where they can be found: template data collection forms; data extracted from included studies; data used for all analyses; analytic code; any other materials used in the review.	33

From: Page MJ, McKenzie JE, Bossuyt PM, Boutron I, Hoffmann TC, Mulrow CD, et al. The PRISMA 2020 statement: an updated guideline for reporting systematic reviews. BMJ 2021;372:n71. doi: 10.1136/bmj.n71. For more information, visit: http://www.prisma-statement.org/

Supplementary Table S2. MEDLINE search strategy.

SET	MEDLINE
1	Bipolar disorder
2	BD
3	Bipolar
4	Manic depressive disorder
5	Manic depressive
6	Manic
7	*Bipolar disorder*
8	Sets 1-7 were combined with "OR"
9	Obsessive-compulsive disorder
10	Obsessive-compulsive
11	OCD
12	*Obsessive-Compulsive Disorder*
13	Sets 9-12 were combined with "OR"
14	Sets 8 and 13 were combined with "AND"
15	Set 14 was limited to May 2022, Humans, English language, Child: 6-12 years, Adolescent: 13-18 years, Adult: 19+ years

Words written in *italic* were used as MeSH headings, the others were used as free text.

Supplementary Table S3. Reference list of papers included in the review.

Previously included references

1. ADAM Y, MEINLSCHMIDT G, GLOSTER AT, LIEB R. Obsessive–compulsive disorder in the community: 12-month prevalence, comorbidity and impairment. Social psychiatry and psychiatric epidemiology. 2012;**47**(3):339-49.
2. ANGST J, GAMMA A, ENDRASS J et al. Obsessive-compulsive severity spectrum in the community: prevalence, comorbidity, and course. European archives of psychiatry and clinical neuroscience. 2004 Jun;**254**(3):156-64.
3. ANGST J, GAMMA A, ENDRASS J et al. Obsessive-compulsive syndromes and disorders: significance of comorbidity with bipolar and anxiety syndromes. European archives of psychiatry and clinical neuroscience. 2005 Feb;**255**(1):65-71.
4. CHEN YW, DILSAVER SC. Comorbidity for obsessive-compulsive disorder in bipolar and unipolar disorders. Psychiatry research. 1995 Nov 29;**59**(1-2):57-64.
5. FARAVELLI C, ABRARDI L, BARTOLOZZI D et al. The Sesto Fiorentino study: background, methods and preliminary results. Lifetime prevalence of psychiatric disorders in an Italian community sample using clinical interviewers. Psychotherapy and psychosomatics. 2004 Jul-Aug;**73**(4):216-25.
6. FIREMAN B, KORAN LM, LEVENTHAL JL, JACOBSON A. The prevalence of clinically recognized obsessive-compulsive disorder in a large health maintenance organization. The American journal of psychiatry. 2001 Nov;**158**(11):1904-10.
7. MERIKANGAS KR, AKISKAL HS, ANGST J et al. Lifetime and 12-month prevalence of bipolar spectrum disorder in the National Comorbidity Survey replication. Archives of general psychiatry. 2007 May;**64**(5):543-52.
8. BOGETTO F, VENTURELLO S, ALBERT U, MAINA G, RAVIZZA L. Gender-related clinical differences in obsessive-compulsive disorder. European psychiatry : the journal of the Association of European Psychiatrists. 1999 Dec;**14**(8):434-41.
9. BOYLAN KR, BIELING PJ, MARRIOTT M, BEGIN H, YOUNG LT, MACQUEEN GM. Impact of comorbid anxiety disorders on outcome in a cohort of patients with bipolar disorder. The Journal of clinical psychiatry. 2004 Aug;**65**(8):1106-13.
10. CANNON DM, ICHISE M, FROMM SJ et al. Serotonin Transporter Binding in Bipolar Disorder Assessed using [11C]DASB and Positron Emission Tomography. Biological psychiatry. 2006;**60**(3):207-17.
11. CASSANO GB, PINI S, SAETTONI M, DELL'OSSO L. Multiple anxiety disorder comorbidity in patients with mood spectrum disorders with psychotic features. The American journal of psychiatry. 1999 Mar;**156**(3):474-6.
12. CENTORRINO F, HENNEN J, MALLYA G, EGLI S, CLARK T, BALDESSARINI RJ. Clinical outcome in patients with bipolar I disorder, obsessive compulsive disorder or both. Human psychopharmacology. 2006 Apr;**21**(3):189-93.

13. COSOFF SJ, HAFNER RJ. The prevalence of comorbid anxiety in schizophrenia, schizoaffective disorder and bipolar disorder. The Australian and New Zealand journal of psychiatry. 1998 Feb;**32**(1):67-72.
14. CRAIG T, HWANG MY, BROMET EJ. Obsessive-compulsive and panic symptoms in patients with first-admission psychosis. The American journal of psychiatry. 2002 Apr;**159**(4):592-8.
15. DELL'OSSO L, PINI S, TUNDO A, SARNO N, MUSETTI L, CASSANO GB. Clinical characteristics of mania, mixed mania, and bipolar depression with psychotic features. Comprehensive psychiatry. 2000 Jul-Aug;**41**(4):242-7.
16. DILSAVER SC, BENAZZI F, AKISKAL KK, AKISKAL HS. Differential patterns of lifetime multiple anxiety disorder comorbidity between Latino adults with bipolar I and major depressive disorders. Bulletin of the Menninger Clinic. 2008 Spring;**72**(2):130-48.
17. DINIZ JB, ROSARIO-CAMPOS MC, SHAVITT RG et al. Impact of age at onset and duration of illness on the expression of comorbidities in obsessive-compulsive disorder. The Journal of clinical psychiatry. 2004 Jan;**65**(1):22-7.
18. EDMONDS LK, MOSLEY BJ, ADMIRAAL AJ et al. Familial bipolar disorder: preliminary results from the Otago Familial Bipolar Genetic Study. The Australian and New Zealand journal of psychiatry. 1998 Dec;**32**(6):823-9.
19. GOES FS, MCCUSKER MG, BIENVENU OJ et al. Co-morbid anxiety disorders in bipolar disorder and major depression: familial aggregation and clinical characteristics of co-morbid panic disorder, social phobia, specific phobia and obsessive-compulsive disorder. Psychological medicine. 2012 Jul;**42**(7):1449-59.
20. HANTOUCHE EG, ANGST J, DEMONFAUCON C, PERUGI G, LANCRENON S, AKISKAL HS. Cyclothymic OCD: a distinct form? Journal of affective disorders. 2003 Jun;**75**(1):1-10.
21. HASLER G, LASALLE-RICCI VH, RONQUILLO JG et al. Obsessive-compulsive disorder symptom dimensions show specific relationships to psychiatric comorbidity. Psychiatry research. 2005 Jun 15;**135**(2):121-32.
22. HENRY C, VAN DEN BULKE D, BELLIVIER F, ETAIN B, ROUILLON F, LEBOYER M. Anxiety disorders in 318 bipolar patients: prevalence and impact on illness severity and response to mood stabilizer. The Journal of clinical psychiatry. 2003 Mar;**64**(3):331-5.
23. ISSLER CK, AMARAL JA, TAMADA RS et al. Clinical expression of obsessive-compulsive disorder in women with bipolar disorder. Revista brasileira de psiquiatria (Sao Paulo, Brazil : 1999). 2005 Jun;**27**(2):139-42.
24. ISSLER CK, MONKUL ES, DE MELLO SIQUEIRA AMARAL JA et al. Bipolar disorder and comorbid obsessive-compulsive disorder is associated with higher rates of anxiety and impulse control disorders. Acta Neuropsychiatrica. 2010;**22**(2):81-6.
25. KOYUNCU A, TUKEL R, OZYILDIRIM I, METERIS H, YAZICI O. Impact of obsessive-compulsive disorder comorbidity on the sociodemographic and clinical features of patients with bipolar disorder. Comprehensive psychiatry. 2010 May-Jun;**51**(3):293-7.

26. KRÜGER S, COOKE RG, HASEY GM, JORNA T. Comorbidity of obsessive compulsive disorder in bipolar disorder. Journal of affective disorders. 1995;**34**(2):117-20.
27. KRUGER S, BRAUNIG P, COOKE RG. Comorbidity of obsessive-compulsive disorder in recovered inpatients with bipolar disorder. Bipolar disorders. 2000 Mar;**2**(1):71-4.
28. LASALLE-RICCI VH, ARNKOFF DB, GLASS CR, CRAWLEY SA, RONQUILLO JG, MURPHY DL. The hoarding dimension of OCD: psychological comorbidity and the five-factor personality model. Behaviour research and therapy. 2006 Oct;**44**(10):1503-12.
29. LENSI P, CASSANO GB, CORREDDU G, RAVAGLI S, KUNOVAC JJ. Obsessive-compulsive disorder. Familial-developmental history, symptomatology, comorbidity and course with special reference to gender-related differences. The British Journal of Psychiatry. 1996;**169**(1):101-7.
30. MAGALHAES PV, KAPCZINSKI NS, KAPCZINSKI F. Correlates and impact of obsessive-compulsive comorbidity in bipolar disorder. Comprehensive psychiatry. 2010 Jul-Aug;**51**(4):353-6.
31. MAHASUAR R, JANARDHAN REDDY YC, MATH SB. Obsessive-compulsive disorder with and without bipolar disorder. Psychiatry and clinical neurosciences. 2011 Aug;**65**(5):423-33.
32. MAINA G, ALBERT U, PESSINA E, BOGETTO F. Bipolar obsessive-compulsive disorder and personality disorders. Bipolar disorders. 2007 Nov;**9**(7):722-9.
33. MARAZZITI D, DELL'OSSO L, DI NASSO E et al. Insight in obsessive-compulsive disorder: a study of an Italian sample. European psychiatry : the journal of the Association of European Psychiatrists. 2002 Nov;**17**(7):407-10.
34. MCELROY SL, STRAKOWSKI SM, KECK PE, JR., TUGRUL KL, WEST SA, LONCZAK HS. Differences and similarities in mixed and pure mania. Comprehensive psychiatry. 1995 May-Jun;**36**(3):187-94.
35. MCELROY SL, ALTSHULER LL, SUPPES T et al. Axis I psychiatric comorbidity and its relationship to historical illness variables in 288 patients with bipolar disorder. The American journal of psychiatry. 2001 Mar;**158**(3):420-6.
36. ORTIZ A, BRADLER K, SLANEY C et al. An admixture analysis of the age at index episodes in bipolar disorder. Psychiatry research. 2011;**188**(1):34-9.
37. PERUGI G, AKISKAL HS, PFANNER C et al. The clinical impact of bipolar and unipolar affective comorbidity on obsessive-compulsive disorder. Journal of affective disorders. 1997 Oct;**46**(1):15-23.
38. PERUGI G, AKISKAL HS, GEMIGNANI A et al. Episodic course in obsessive-compulsive disorder. European archives of psychiatry and clinical neuroscience. 1998;**248**(5):240-4.

39. PERUGI G, AKISKAL HS, RAMACCIOTTI S et al. Depressive comorbidity of panic, social phobic, and obsessive-compulsive disorders re-examined: is there a bipolar II connection? Journal of psychiatric research. 1999 Jan-Feb;**33**(1):53-61.
40. PERUGI G, TONI C, FRARE F, TRAVIERSO MC, HANTOUCHE E, AKISKAL HS. Obsessive-compulsive-bipolar comorbidity: a systematic exploration of clinical features and treatment outcome. The Journal of clinical psychiatry. 2002 Dec;**63**(12):1129-34.
41. PINI S, CASSANO GB, SIMONINI E, SAVINO M, RUSSO A, MONTGOMERY SA. Prevalence of anxiety disorders comorbidity in bipolar depression, unipolar depression and dysthymia. Journal of affective disorders. 1997 Feb;**42**(2-3):145-53.
42. PINI S, DELL'OSSO L, MASTROCINQUE C et al. Axis I comorbidity in bipolar disorder with psychotic features. The British journal of psychiatry : the journal of mental science. 1999 Nov;**175**:467-71.
43. SAUNDERS EF, FITZGERALD KD, ZHANG P, MCINNIS MG. Clinical features of bipolar disorder comorbid with anxiety disorders differ between men and women. Depression and anxiety. 2012 Aug;**29**(8):739-46.
44. SHABANI A, ALIZADEH A. The specific pattern of obsessive-compulsive symptoms in patients with bipolar disorder. Journal of Research in Medical Sciences. 2008;**13**(2):48-54.
45. SIMON NM, SMOLLER JW, FAVA M et al. Comparing anxiety disorders and anxiety-related traits in bipolar disorder and unipolar depression. Journal of psychiatric research. 2003 May-Jun;**37**(3):187-92.
46. SIMON NM, OTTO MW, WISNIEWSKI SR et al. Anxiety disorder comorbidity in bipolar disorder patients: data from the first 500 participants in the Systematic Treatment Enhancement Program for Bipolar Disorder (STEP-BD). The American journal of psychiatry. 2004 Dec;**161**(12):2222-9.
47. STRAKOWSKI SM, TOHEN M, STOLL AL, FAEDDA GL, GOODWIN DC. Comorbidity in mania at first hospitalization. American Journal of Psychiatry. 1992;**149**(4):554-6.
48. STRAKOWSKI SM, SAX KW, MCELROY SL, KECK PE, JR., HAWKINS JM, WEST SA. Course of psychiatric and substance abuse syndromes co-occurring with bipolar disorder after a first psychiatric hospitalization. The Journal of clinical psychiatry. 1998 Sep;**59**(9):465-71.
49. TAMAM L, OZPOYRAZ N. Comorbidity of anxiety disorder among patients with bipolar I disorder in remission. Psychopathology. 2002 Jul-Aug;**35**(4):203-9.
50. TIMPANO KR, RUBENSTEIN LM, MURPHY DL. Phenomenological features and clinical impact of affective disorders in OCD: a focus on the bipolar disorder and OCD connection. Depression and anxiety. 2012 Mar;**29**(3):226-33.
51. TUKEL R, METERIS H, KOYUNCU A, TECER A, YAZICI O. The clinical impact of mood disorder comorbidity on obsessive-compulsive disorder. European archives of psychiatry and clinical neuroscience. 2006 Jun;**256**(4):240-5.

52. TUKEL R, OFLAZ SB, OZYILDIRIM I et al. Comparison of clinical characteristics in episodic and chronic obsessive-compulsive disorder. Depression and anxiety. 2007;**24**(4):251-5.
53. ZUTSHI A, REDDY YC, THENNARASU K, CHANDRASHEKHAR CR. Comorbidity of anxiety disorders in patients with remitted bipolar disorder. European archives of psychiatry and clinical neuroscience. 2006 Oct;**256**(7):428-36.
54. ZUTSHI A, KAMATH P, REDDY YC. Bipolar and nonbipolar obsessive-compulsive disorder: a clinical exploration. Comprehensive psychiatry. 2007 May-Jun;**48**(3):245-51.
55. DILSAVER SC, AKISKAL HS, AKISKAL KK, BENAZZI F. Dose-response relationship between number of comorbid anxiety disorders in adolescent bipolar/unipolar disorders, and psychosis, suicidality, substance abuse and familiality. Journal of affective disorders. 2006 Dec;**96**(3):249-58.
56. JOSHI G, MICK E, WOZNIAK J et al. Impact of obsessive-compulsive disorder on the antimanic response to olanzapine therapy in youth with bipolar disorder. Bipolar disorders. 2010 Mar;**12**(2):196-204.
57. JOSHI G, WOZNIAK J, PETTY C et al. Clinical characteristics of comorbid obsessive-compulsive disorder and bipolar disorder in children and adolescents. Bipolar disorders. 2010 Mar;**12**(2):185-95.
58. MASI G, PERUGI G, TONI C et al. Obsessive-compulsive bipolar comorbidity: Focus on children and adolescents. Journal of affective disorders. 2004;**78**(3):175-83.
59. MASI G, MILLEPIEDI S, MUCCI M, BERTINI N, MILANTONI L, ARCANGELI F. A naturalistic study of referred children and adolescents with obsessive-compulsive disorder. Journal of the American Academy of Child and Adolescent Psychiatry. 2005 Jul;**44**(7):673-81.
60. MASI G, PERUGI G, MILLEPIEDI S et al. Bipolar co-morbidity in pediatric obsessive-compulsive disorder: clinical and treatment implications. Journal of child and adolescent psychopharmacology. 2007 Aug;**17**(4):475-86.
61. MASI G, MILLEPIEDI S, PERUGI G et al. Pharmacotherapy in paediatric obsessive-compulsive disorder: a naturalistic, retrospective study. CNS drugs. 2009;**23**(3):241-52.
62. MASI G, MILLEPIEDI S, PERUGI G et al. A naturalistic exploratory study of the impact of demographic, phenotypic and comorbid features in pediatric obsessive-compulsive disorder. Psychopathology. 2010;**43**(2):69-78.
63. REDDY YC, REDDY PS, SRINATH S, KHANNA S, SHESHADRI SP, GIRIMAJI SC. Comorbidity in juvenile obsessive-compulsive disorder: a report from India. Canadian journal of psychiatry Revue canadienne de psychiatrie. 2000 Apr;**45**(3):274-8.

64. TILLMAN R, GELLER B, BOLHOFNER K, CRANEY JL, WILLIAMS M, ZIMERMAN B. Ages of onset and rates of syndromal and subsyndromal comorbid DSM-IV diagnoses in a prepubertal and early adolescent bipolar disorder phenotype. Journal of the American Academy of Child and Adolescent Psychiatry. 2003 Dec;**42**(12):1486-93.

New references

1. ABRAMOVITCH A, ANHOLT GE, COOPERMAN A, et al. Body mass index in obsessive-compulsive disorder. J Affect Disord 2019; **245**:145–51.
2. ALVARENGA PG, DO ROSARIO MC, CESAR RC, et al. Obsessive–compulsive symptoms are associated with psychiatric comorbidities, behavioral and clinical problems: a population-based study of Brazilian school children. Eur Child Adolesc Psychiatry 2016; **25**:175–82.
3. ANHOLT GE, ADERKA IM, VAN BALKOM AJLM, et al. Age of onset in obsessive–compulsive disorder: admixture analysis with a large sample. Psychol Med 2014; **44**:185–94.
4. BAPTISTA T, GALUÉ L, MARTÍNEZ F. Bidirectional comorbidity between bipolarand obsessive-compulsive disorders: symptoms frequency, treatment challenges and underexplored areas. Invest Clin 2020; **61**:189–95.
5. BENATTI B, DELL'OSSO B, ARICI C, HOLLANDER E, ALTAMURA AC. Characterizing impulsivity profile in patients with obsessive–compulsive disorder. Int J Psychiatry Clin Pract 2014; **18**:156–60.
6. BENER A, DAFEEAH EE, ABDULLA M AL, ABOU-SALEH MT, VENTRIGLIO A. Comorbid obsessive-compulsive disorder and bipolar disorder in a highly endogamous population: which came first? Int J Cult Ment Health 2016; **9**:407–13.
7. BERKOL TD, AYTAC HM. Comparison of Clinical Features of Bipolar Disorder Patients with and without Psychiatric Comorbidity. Eurasian J Med 2021; **53**:203–7.
8. BRAMANTE S, QUARATO F, MEHANOVIĆ E, RIGARDETTO S, MAINA G. The forbidden thoughts dimension and psychiatric comorbidities in a large sample of OCD patients: A possible link to bipolar I comorbid disorder. J Obsessive Compuls Relat Disord 2021; **29**:100642.
9. BRAVERMAN L, FUCHS C, WEIZMAN A, POYUROVSKY M. Elevated rate of OCD-spectrum and tic disorders in patients with bipolar depression and comorbid OCD. J Obsessive Compuls Relat Disord 2021; **29**:100643.
10. BRAVERMAN L, FUCHS C, WEIZMAN A, POYUROVSKY M. Rate of OCD and sub-threshold OCD in bipolar disorder patients with first depressive episode. Psychiatry Res 2021; **302**:114010.

11. CARTA MG, FINEBERG N, MORO MF, et al. The Burden of Comorbidity Between Bipolar Spectrum and Obsessive-Compulsive Disorder in an Italian Community Survey. Front Psychiatry 2020;**11**.
12. CEDERLÖF M, LICHTENSTEIN P, LARSSON H, et al. Obsessive-Compulsive Disorder, Psychosis, and Bipolarity: A Longitudinal Cohort and Multigenerational Family Study. Schizophr Bull 2015; **41**:1076–83.
13. DAS A. Anxiety Disorders in Bipolar I Mania: Prevalence, Effect on Illness Severity, and Treatment Implications. Indian J Psychol Med 2013; **35**:53–9.
14. DE FILIPPIS R, ALOI M, BRUNI A, GAETANO R, SEGURA-GARCIA C, DE FAZIO P. Bipolar disorder and obsessive compulsive disorder: The comorbidity does not further impair the neurocognitive profile. J Affect Disord 2018; **235**:1–6.
15. DI SALVO G, PESSINA E, ARAGNO E, et al. Impact of comorbid obsessive-compulsive disorder on suicidality in patients with bipolar disorder. Psychiatry Res 2020; **290**:113088.
16. DOMINGUES-CASTRO MS, TORRESAN RC, SHAVITT RG, et al. Bipolar disorder comorbidity in patients with obsessive-compulsive disorder: Prevalence and predictors. J Affect Disord 2019; **256**:324–30.
17. FINEBERG NA, HENGARTNER MP, BERGBAUM C, GALE T, RÖSSLER W, ANGST J. Lifetime comorbidity of obsessive-compulsive disorder and sub-threshold obsessive-compulsive symptomatology in the community: impact, prevalence, socio-demographic and clinical characteristics. Int J Psychiatry Clin Pract 2013; **17**:188–96.
18. HOFER PD, WAHL K, MEYER AH, et al. Obsessive-compulsive disorder and the risk of subsequent mental disorders: A community study of adolescents and young adults. Depress Anxiety 2018; **35**:339–45.
19. HUANG L-C, TSAI K-J, WANG H-K, ET AL. Prevalence, incidence, and comorbidity of clinically diagnosed obsessive–compulsive disorder in Taiwan: A national population-based study. Psychiatry Res 2014; **220**:335–41.
20. JAKUBOVSKI E, DINIZ JB, VALERIO C, et al. Clinical predictors of long-term outcome in obsessive-compulsive disorder. Depress Anxiety 2013; **30**:763–72.
21. JEON S, BAEK JH, YANG SY, et al. Exploration of comorbid obsessive-compulsive disorder in patients with bipolar disorder: The clinic-based prevalence rate, symptoms nature and clinical correlates. J Affect Disord 2018; **225**:227–33.
22. KAZHUNGIL F, CHOLAKOTTIL A, KATTUKULATHIL S, KOTTELASSAL A, VAZHAKALAYIL R. Clinical and familial profile of bipolar disorder with and without obsessive-compulsive disorder: an Indian study. Trends Psychiatry Psychother 2017; **39**:270–5.
23. KEMP DE, SYLVIA LG, CALABRESE JR, et al. General medical burden in bipolar disorder: findings from the LiTMUS comparative effectiveness trial. Acta Psychiatr Scand 2014; **129**:24–34.

24. KHAN Q UL AIN, YOUNUS S, HASAN H, KHAN MZ. Association of bipolar I disorder with obsessive compulsive disorder: A clinical study from Pakistan. Neurol Psychiatry Brain Res 2019; **33**:89–92.
25. KHORSHIDIAN F, HAMIDIA A, KHEIRKHAH F, et al. Comparison of Aripiprazole and Risperidone effectiveness in treating obsessive-compulsive disorder in patients with bipolar disorder: Double-blind, randomized clinical trial. Heal Sci Reports 2023; **6**.
26. KIM S-W, BERK L, KULKARNI J, et al. Impact of comorbid anxiety disorders and obsessive-compulsive disorder on 24-month clinical outcomes of bipolar I disorder. J Affect Disord 2014; **166**:243–8.
27. MASI G, BERLOFFA S, MUCCI M, et al. A naturalistic exploratory study of obsessive-compulsive bipolar comorbidity in youth. J Affect Disord 2018; **231**:21–6.
28. OZDEMIROGLU F, SEVINCOK L, SEN G, et al. Comorbid obsessive–compulsive disorder with bipolar disorder: A distinct form? Psychiatry Res 2015; **230**:800–5.
29. PAUL I, SINHA VK, SARKHEL S, PRAHARAJ SK. Co-morbidity of Obsessive-compulsive Disorder and Other Anxiety Disorders with Child and Adolescent Mood Disorders. East Asian Arch Psychiatry 2015; **25**:58–63.
30. SAHRAIAN A, JAHROMI LR, GHANIZADEH A, MOWLA A. Memantine as an Adjuvant Treatment for Obsessive Compulsive Symptoms in Manic Phase of Bipolar Disorder. J Clin Psychopharmacol 2017; 37:246–9.
31. SAHRAIAN A, EHSAEI Z, MOWLA A. Aripiprazole as an adjuvant treatment for obsessive and compulsive symptoms in manic phase of bipolar disorder: A randomized, double-blind, placebo-controlled clinical trial. Prog Neuro-Psychopharmacology Biol Psychiatry 2018; **84**:267–71.
32. SAHRAIAN A, GHAHREMANPOURI B, MOWLA A. Is quetiapine effective for obsessive and compulsive symptoms in patients with bipolar disorder? A randomized, double-blind, placebo-controlled clinical trial. CNS Spectr 2022; **27**:634–8.
33. SARAF G, PAUL I, VISWANATH B, NARAYANASWAMY JC, MATH SB, REDDY YCJ. Bipolar disorder comorbidity in patients with a primary diagnosis of OCD. Int J Psychiatry Clin Pract 2016; 1–5.
34. SHASHIDHARA M, SUSHMA BR, VISWANATH B, MATH SB, JANARDHAN REDDY YC. Comorbid obsessive compulsive disorder in patients with bipolar-I disorder. J Affect Disord 2015; **174**:367–71.
35. SUBRAMANIAM M, ABDIN E, VAINGANKAR J, et al. Obsessive-Compulsive Disorder in Singapore: Prevalence, Comorbidity, Quality of Life and Social Support. Ann Acad Med Singapore 2020; **49**:15–25.
36. TASDEMIR A, TAMAM L, KESKIN N, EVLICE YE. Assessment of co-morbidity of adult separation anxiety in patients with bipolar disorder. Nord J Psychiatry 2016; **70**:93–102.

37. TEH WL, ABDIN E, VAINGANKAR J, et al. Prevalence and correlates of bipolar spectrum disorders in Singapore: Results from the 2016 Singapore Mental Health Study (SMHS 2016). J Affect Disord 2020; **274**:339–46.
38. TONNA M, TRINCHIERI M, LUCARINI V, et al. Pattern of occurrence of obsessive-compulsive symptoms in bipolar disorder. Psychiatry Res 2021; **297**:113715.
